# Tumor Necrosis Factor Superfamily: Ancestral Functions and Remodeling in Early Vertebrate Evolution

**DOI:** 10.1093/gbe/evaa140

**Published:** 2020-07-06

**Authors:** Ignacio Marín

**Affiliations:** Instituto de Biomedicina de Valencia, Consejo Superior de Investigaciones Científicas (IBV-CSIC), Valencia, Spain

**Keywords:** tumor necrosis factor, early vertebrate evolution, immune system, Ligand-receptor coevolution

## Abstract

The evolution of the tumor necrosis factor superfamily (TNFSF) in early vertebrates is inferred by comparing the TNFSF genes found in humans and nine fishes: three agnathans, two chondrichthyans, three actinopterygians, and the sarcopterygian *Latimeria chalumnae*. By combining phylogenetic and synteny analyses, the TNFSF sequences detected are classified into five clusters of genes and 24 orthology groups. A model for their evolution since the origin of vertebrates is proposed. Fifteen TNFSF genes emerged from just three progenitors due to the whole-genome duplications (WGDs) that occurred before the agnathan/gnathostome split. Later, gnathostomes not only kept most of the genes emerged in the WGDs but soon added several tandem duplicates. More recently, complex, lineage-specific patterns of duplications and losses occurred in different gnathostome lineages. In agnathan species only seven to eight TNFSF genes are detected, because this lineage soon lost six of the genes emerged in the ancestral WGDs and additional losses in both hagfishes and lampreys later occurred. The orthologs of many of these lost genes are, in mammals, ligands of death-domain-containing TNFSF receptors, indicating that the extrinsic apoptotic pathway became simplified in the agnathan lineage. From the patterns of emergence of these genes, it is deduced that both the regulation of apoptosis and the control of the NF-κB pathway that depends in modern mammals on TNFSF members emerged before the ancestral vertebrate WGDs.

## Introduction

Cell signaling is crucial for the development of multicellular organisms. In metazoans, one of the most important signaling systems is based on the interactions among ligands of the tumor necrosis factor superfamily (TNFSF) and their receptors, collectively known as the tumor necrosis factor receptor superfamily (TNFRSF; Locksley et al. 2001; Aggarwal 2003; Hehlgans and Pfeffer 2005; Aggarwal et al. 2012; Dostert et al. 2019). The number of genes encoding TNFSF and TNFRSF proteins varies substantially among species but is often large. For instance, humans have 18 genes encoding TNFSF ligands and 29 genes encoding TNFRSFs (Aggarwal 2003; Dostert et al. 2019). Given this multiplicity, it can be expected that they participate in many different processes. In mammals, the set of TNFSF/TNFRSF interacting pairs (shortened from now on as “TNFSF/TNFRSF system”) has fundamental roles in the development and function of the immune system and additional essential roles regulating apoptosis, cell survival and proliferation, cell differentiation, and organogenesis (Locksley et al. 2001; Aggarwal 2003; Hehlgans and Pfeffer 2005; Wallach 2018; Yi et al. 2018; Dostert et al. 2019). Some of these roles are ancient; the invertebrate TNFSF/TNFRSF system is also involved in apoptosis, immune defense, and control of cell number (Igaki and Miura 2014; Quistad et al. 2014; Qin et al. 2019). In our species, mutations in TNFSF ligands and TNFRs have been shown to cause congenital syndromes (Locksley et al. 2001; Lobito et al. 2011). The TNFSF/TNFRSF system also influences the likelihood of suffering inflammatory diseases, autoimmune diseases, and cancer (Locksley et al. 2001; Aggarwal et al. 2012).

Characterizing all the TNFSF genes present in a given genome is relatively easy because they all encode proteins that have a characteristic C-terminal module known as TNF homology domain (THD). The amino acid sequence of the THD is sufficiently conserved as to allow detecting the TNFSF genes present in any given species by conventional similarity searches. It also allows for long-range evolutionary analyses of the TNF superfamily. For example, orthologs can be characterized in organisms as distant as mammals and fishes (Glenney and Wiens 2007; Biswas et al. 2015). However, orthologies among vertebrate and nonvertebrate genes are far less clear, with sequence-based tree topologies systematically suffering from poor statistical support (Robertson et al. 2006; Huang et al. 2008; Zhang et al. 2008, 2010; Pozzolini et al. 2016; Redmond et al. 2017; see discussion in Wiens and Glenney 2011). Almost all studied TNFSF/TNFRSF functional interactions involve ligand and receptor trimers, although exceptional higher order structures have been also described (Zhang 2004). THD conservation is due to its involvement in both formation of ligand trimers and in ligand-receptor interaction and specificity, which is established when the THD contacts peculiar cysteine-rich domains present in the TNFRs (Bodmer et al. 2002; Zhang 2004; Vanamee and Faustman 2018). Most TNFSF proteins are type II transmembrane proteins but many of them appear both as membrane-linked and as soluble forms, the latter produced after cleavage by metalloproteases of the membrane-anchored proteins. The different local concentrations and affinities of the membrane-bound and soluble proteins further increase the flexibility of the TNFSF/TNFRSF system (Wallach 2018).

The evolution of the TNF superfamily has been quite extensively analyzed. A summary of the most interesting findings is as follows: 1) This superfamily is restricted to metazoans. Most animals, including sponges (Pozzolini et al. 2016), the sister group of all other animals (Feuda et al. 2017; Zhao et al 2019) have at least one TNFSF gene, indicating that the TNFSF/TNFRSF system arose very early in animal evolution. Exceptionally, some metazoans, for example, nematodes such as *Caenorhaditis elegans*, lack TNFSF genes (Ruvkun and Hobert 1998). However, given that they are present in other ecdysozoans, such as arthropods, this is clearly a secondary loss. 2) When present, the number of genes is highly variable, ranging from 1, as in the fly *Drosophila melanogaster* (Moreno et al. 2002) to as many as 23 in the mollusk *Crassostrea gigas* (Gao et al. 2015) or 24 in the cephalochordate *Branchiostoma floridae* (Huang et al. 2008). 3) There is no clear relationship between complexity and number of TNFSF genes; a relatively simple organism as the coral *Acropora digitifera* has more genes than some vertebrate species (Quistad et al. 2014). 4) Extensive remodeling of the TNFSF/TNFRSF system may occur in relatively short evolutionary times. For example, although *Branchiostoma* and vertebrates have many TNFSF genes, the urochordate *Ciona intestinalis* only has four (Parrinello et al. 2018). Given that *Ciona* is evolutionary closer to vertebrates than *Branchiostoma* (Delsuc et al. 2006, 2008, 2018), either two large independent amplifications occurred in the lineages that gave rise to cephalochordates and vertebrates or a drastic reduction occurred in the *Ciona* lineage. 5) The two whole-genome duplications (WGDs) that occurred just after the split that separated vertebrates from the rest of chordates (Kuraku et al. 2009; Smith et al. 2013; Sacerdot et al. 2018) had a significant role in increasing the number of TNFSF genes in the vertebrate lineage. This was first shown by demonstrating that some TNFSF genes are linked to the MHC regions known to be quadruplicated because of those two WGDs (Kasahara 1998; Kaufman 2018). 6) Tandem duplications have also had a significant role in increasing the number of TNFSF genes, as shown by the presence of very similar TNFSF genes in tandem in many species (Huang et al. 2008; Wiens and Glenney 2011). 7) In vertebrates, there is evidence for coevolution of the TNF and TNFR superfamilies, that is, an increase in the number of ligands correlates with an increase in the number of receptors, in such a way that the new ligands generally bind to the receptors arisen at about the same time (Collette et al. 2003; see also Beutler and Van Huffel 1994).

Despite all this information, there are still several weak points in our knowledge. One of the most significant concerns the evolution of the TNFSF/TNFRSF system in early vertebrates. The precise patterns of evolution of the TNF superfamily have been studied in a few vertebrate groups such as mammals, teleosts, and birds (Collette et al. 2003; Kaiser et al. 2005; Savan et al. 2005; Glenney and Wiens 2007; Dalloul et al. 2010; Hong et al. 2013; Biswas et al. 2015; Premzl 2016; Rohde et al. 2018). However, very significant groups, such as chondrichthyans and agnathans, have been so far neglected. Only some TNFSF genes have been described in sharks (Pantalacci et al. 2008; Ren et al. 2011; Li et al. 2012, 2015; Venkatesh et al. 2014) and data for lampreys or hagfishes are even more limited (Suzuki et al. 2004; Das et al. 2016). In this work, an exhaustive analysis is performed comparing the human TNFs with those in nine fish species. The results obtained extend our knowledge of the early evolution of the TNF superfamily in vertebrates. A precise model for the early evolution of the TNF superfamily, which accounts for the impact of whole-genome and tandem duplications and the importance of gene loss in each lineage, is developed. From this model, conclusions on the early functions of the TNFSF/TNFRSF system are obtained.

## Results

### General Characterization of the TNF Superfamily in Fish Model Species

A deep, iterative search for TNF genes in selected fish species was performed using TBlastN analyses (see Materials and Methods). The total number of TNF superfamily genes found in the nine fish species analyzed was as follows: eight in *Eptatretus burgeri*, eight in *Lethenteron camtschaticum*, seven in *Petromyzon marinus*, 29 in *Rhindocon typus*, 20 in *Callorhinchus milii*, 19 in *Danio rerio*, 14 in *Takifugu rubripes*, 20 in *Lepisosteus oculatus*, and 25 in *Latimeria chalumnae.* As described above, our species has 18 TNFSF genes, so it is not difficult to find fishes with a more complex TNFSF set. In fact, the *Rhincodon* TNFSF gene set found here is the largest so far described in any organism. These results also suggest that either a substantial increase of genes occurred in the gnathostomes or a significant reduction in agnathans. The number of TNFSF genes detected in some species was unexpected. Similar searches by Glenney and Wiens (2007) found only 18 TNFSF sequences in *Danio* and 11 in *Takifugu*, whereas Biswas et al. (2015) detected 16 in *Danio* and 12 in *Takifugu*. Similarly, Venkatesh et al. (2014) found just five TNFSF sequences in *Callorhinchus* and Tacchi et al. (2015) only 14 in *Latimeria*. These discrepancies may be explained by the analyzed genomes not being fully sequenced at the time when those previous searches were performed, although it is also possible that our detection methods have been more exhaustive. Another unexpected finding is that the spotted gar *Lepisosteus* has more TNFSF genes than *Danio* or *Takifugu*, despite an additional, teleost-specific WGD that could have increased the number of TNFSF genes in the two latter species (Braasch et al. 2016).

### TNFSF Genes and Orthology Groups in Gnathostomes

**Fig. 1:**
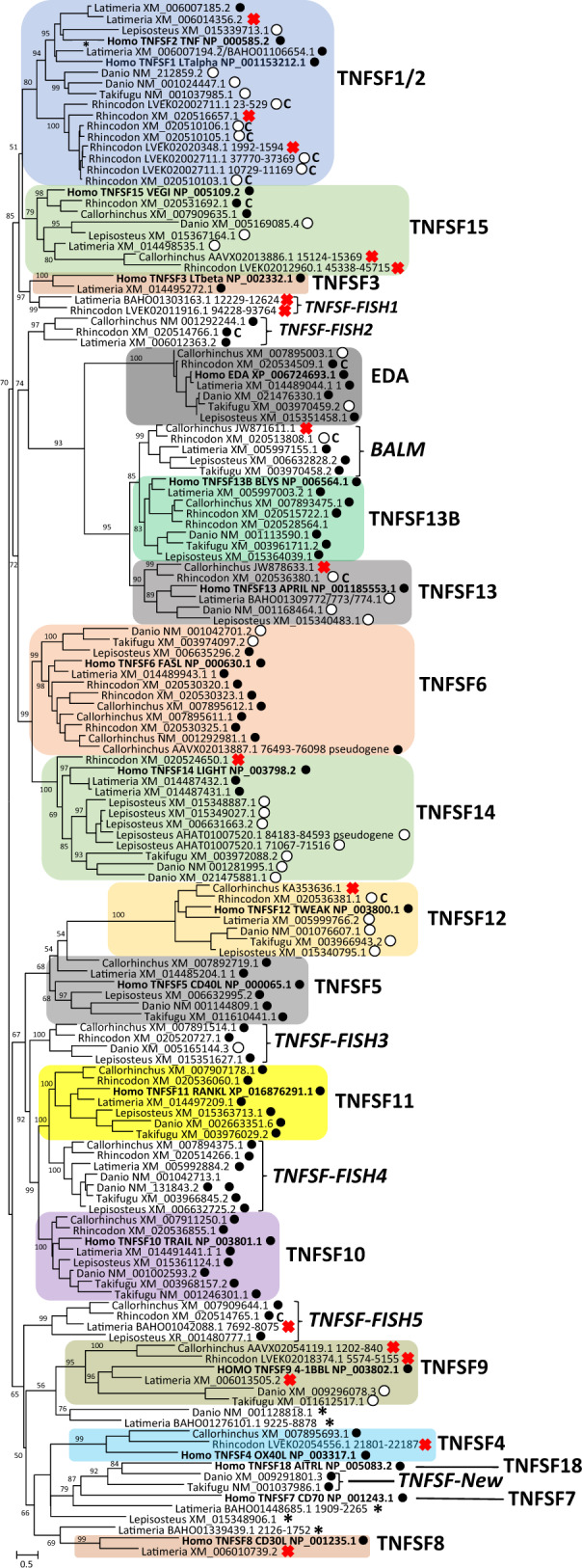
ML tree based on TNFSF sequences in gnathostome species. The tree was obtained from an alignment (supplementary file 1, Supplementary Material online) of their THDs. Individual sequences are identified by genus name and accession number. The 24 orthology groups detected are also indicated (right, in large letters; notice that TNFSF7 and TNFSF18 only include a single human sequence). When the accession number refers to a genomic contig, the precise location of the THD is indicated with numbers that correspond to the start and the end of the region in which similarity was detected. When two sequences are indicated (separated by a slash), it means that the final THD sequence was built taking into account the information provided by both sequences. For the 17 orthology groups that include human genes, it was determined whether the TNFSF genes in other species are either located in the same position (i.e., are surrounded by the same genes) that their human orthologs (black dots) or surrounded by different genes that are however located in the human chromosome where that putative TNFSF ortholog is found (white dots). For six orthology groups that do not include human genes, synteny was established taken the genomes of fish species as references. The species taken as starting point was chosen depending on the available data: *Latimeria* (for TNFSF-Fish2, BALM, and TNFSF-Fish4), *Lepisosteus* (for TNFSF-Fish3 and TNFSF-Fish5) or *Danio* (TNFSF-New). The meaning of the black and white dots in these six cases is the same as just indicated but respect to the reference fish species. For TNFSF-Fish1 genes, synteny was impossible to determine given the available data. Bootstrap values (in percentages) are indicated as numbers adjacent to the corresponding branches. Values for external, recent branches of the tree or those below 50% have been omitted for simplicity. Crosses indicate lack of genomic data that prevent the synteny analyses. In 13 cases, a capital C adjacent to the white or black dots indicates that synteny data are based on the *Carcharodon carcharias* orthologs of the corresponding *Rhincodon* genes. *Carcharodon* was used given that it is a close relative of *Rhincodon* and some regions are better assembled in the *Carcharodon* than in the *Rhincodon* genome (see Materials and Methods).

Figure 1 shows the maximum-likelihood (ML) phylogenetic tree obtained from the 145 aligned gnathostome sequences. The corresponding alignment can be found in supplementary file 1, Supplementary Material online. Synteny results have been also incorporated into the tree and shown in the same figure. From now on, the genes in fishes will be named according to the accession numbers of their sequences. Although this is somewhat unusual, we found that using the gene symbols assigned in the corresponding genome projects would cause all kinds of confusions because many of the names assigned are misleading (e.g., genes called with the same name in different species often have no relationship; obvious orthologs often receive different names, etc.).The topology shown in figure 1 agrees well with that found by other authors with related data sets (Glenney and Wiens 2007; Biswas et al. 2015). The main difference refers to the position of *TNFSF5* and *TNFSF12*, which in other trees appeared in several alternative positions (Kaiser et al. 2005; Glenney and Wiens 2007; Biswas et al. 2015). However, the location observed for *TNFSF5* and *TNFSF12* in figure 1, that is, close to *TNFSF10* and *TNFSF11*, is precisely the one expected according to the patterns of WGD duplications, which suggested that these four genes come from a common ancestor (Collette et al. 2003). Similarly, synteny data shown in figure 1, which are detailed in supplementary file 2, Supplementary Material online, fully confirm those found by Glenney and Wiens (2007) for *Homo*, *Danio*, and *Takifugu*, extending those findings to the other fish species. As it is obvious in figure 1, synteny results were in general congruent (i.e., multiple fish species provided identical results) and agreed with the topology of the tree (i.e., very similar sequences that group together in fig. 1 are often in the same exact positions or at least in the same chromosomes in different species). Finally, the results in figure 1 agree quite well with previous works (Glenney and Wiens 2007; Tacchi et al. 2015) respect to which human genes are present or absent in fishes. However, some differences have been detected. Thus, Glenney and Wiens (2007) described the lack of *TNFSF3*, *-4*, *-7*, *-8*, *-18*, and possibly *TNFSF15* in fishes. However, in figure 1, likely fish orthologs for all those genes except *TNFSF7* and *TNFSF18* are detailed. This is largely due to our analysis including additional species.

By combining information from the topology of the tree, supported by statistical bootstrap values, and the conserved synteny data summarized in figure 1, sequences were classified into 24 orthology groups, seven of them absent in humans (*BALM*, *TNFSF-Fish1* to *-Fish5*, and *TNFSF-New*). For obvious reasons, these groups include both orthologs in different species and species-specific recent paralogs with very similar sequences. In total, 140 of the 145 sequences in the tree are included in one of these 24 groups; the exceptions are indicated with asterisks in figure 1. For the human *TNFSF7/CD70* (aka *CD27L*) and *TNFSF18/AITRL* genes, no obvious orthologs were found in fishes (fig. 1). These are the only orthology groups defined that contain just one gene. Only in a few cases, significant (≥95%) bootstrap support was found for internal branches of the tree that include two or more orthology groups. This means that sequence similarity by itself is often insufficient to determine ancient relationships among TNFSF genes. However, combining sequence similarity, synteny data and knowledge about the timing of duplication events (e.g., ancient WGDs vs. more recent tandem duplications), is sometimes possible to go much back in time than by sequence analyses alone. Collette et al. (2003) proposed that all TNFSF human genes are located in eight particular chromosomes because they derive from two clusters of genes present in different chromosomes in the vertebrate ancestor that become multiplied by four due to the two rounds of WGD occurred in early vertebrate evolution. The first of the chromosomal quartets (in humans, chromosomes 1, 6, 9, and 19) corresponds to the well-supported MHC paralogons, already cited above (Kasahara 1998). We have confirmed their results and determined that all these regions derived from the ancestral chromosome 9 deduced by Sacerdot et al. (2018). Collette et al. (2003) also proposed that the TNFSF-containing regions in human chromosomes 3, 13, 17, and X are also paralogons. At that time, evidence for this hypothesis was quite weak, namely the similarity of some TNFSF genes, such as *EDA*, *TNFSF13*, and *TNFSF13B* or *TNFSF10*, *TNFSF11*, and *TNFSF5* (Collette et al. 2003). Today, much more information is available and the hypothesis can be easily tested. Indeed, totally supporting Collette et al. (2003) suggestion, the regions where all these genes are found can be traced back to a single ancestral pre-WGD chromosome (number 16, according to the nomenclature proposed by Sacerdot et al. [2018]).

### The TNF Cluster: TNFSF1/2/3, TNFSF15, and Related Fish Genes

Here and in the next sections, we will use all the available information to divide the TNF superfamily into five clusters of genes (TNF, EDA, FASL, CD40L, and 4-1BBL clusters), all of them emerged very early in vertebrate evolution.

Going from top to bottom in figure 1, the first interesting finding is that two human genes, *TNFSF1/LTα* and *TNFSF2/TNF*, are so similar that they must be included into a single assembly, named from now on TNFSF1/2 orthology group. The close association in phylogenetic trees of those two genes was also detected in earlier works (Collette et al. 2003; Glenney and Wiens 2007; Kinoshita et al. 2014; Biswas et al. 2015; Premzl 2016). In our species, these two genes are located in a tandem (at 6p21.33; Browning et al. 1993) that also includes *TNFSF3/LTβ*. A set of bony fish sequences very similar to human *TNFSF1/LTα* and *TNFSF2/TNF* (an association supported by high bootstrap results; fig. 1) were found. Given the topology observed for the TNFSF1/2 group, it must be considered that all of them, one to three per species, are equally related to human *TNFSF1/LTα* and *TNFSF2/TNF*, that is, no orthology can be ascribed by sequence similarity alone. However, synteny may complement sequence data to obtain further insights. As already mentioned, human *TNFSF1/LTα*, *TNFSF2/TNF*, and *TNFSF3/LTβ* are located in tandem. The exact same relative disposition and orientation observed in humans has been detected for three genes found in *Latimeria*, in such a way that the TNFSF1/2 group gene XM_00607194.2 (wrongly called “TNFSF15” in the *Latimeria* genome annotation) would correspond to human *TNFSF1/LTα*, a second TNFSF1/2 gene, XM_006007185.2 (LOC102357196 in the *Latimeria* genome annotation), would be the *TNFSF2/TNF* ortholog and XM_014495272.1 (aka LOC102357462) would correspond to *TNFSF3/LTβ*. Notice that this last association is strongly supported by bootstrap analyses (fig. 1, TNFSF3 group). Such cluster is absent in actinopterygians, as first described by Glenney and Wiens (2007) and confirmed here. However, four TNFSF1/2 genes in *Danio* (two genes*)*, *Takifugu*, and *Lepisosteus* are surrounded by genes located in humans in the 6p21 region (white dots in fig. 1 and supplementary file 2, Supplementary Material online). The two *Danio* genes are most likely the result of the teleost-specific WGD (see details in supplementary file 2, Supplementary Material online). *Latimeria* has a third TNFSF1/2 gene (accession number XM_006014356.2), but its exact location could not be determined, because it is found in a hitherto isolated contig.

Bootstrap support for the branch that puts eight *Rhincodon* TNFSF1/2-like sequences together with the rest of TNFSF1/2 sequences is low (80%). That these may correspond to true orthologs of human *TNFSF1/LTα* and/or *TNFSF2/TNF* is supported by synteny data. Six *Rhincodon* sequences (those with white dots in fig. 1) are located in tandem. Although the region of the *Rhincodon* genome that contains this tandem is not fully assembled, the same tandem is observed in the genome of the closely related shark *Carcharodon carcharias* (accession number QUOW01004891.1) and it turns out that the orthologs of the genes that surround this *Carcharodon* TNFSF tandem are found in humans in region 6p22, close to where the human TNFSF1/2/3 tandem is located (supplementary file 2, Supplementary Material online).

In summary, the results obtained for the TNFSF1/2 and TNFSF3 orthology groups are compatible with a single progenitor of all these genes being present in early vertebrates that later became several times independently duplicated in tandem in different lineages. Given its position in the tree and phylogenetic span, *TNFSF3/LTβ* genes may be interpreted as rapidly evolving duplicates of either *TNFSF1/LTα* or *TNFSF2/TNF*, emerged after the actinopterygian/sarcopterygian split. *Danio* NM_00102447.1 (called *TNFB* in the *Danio* genome annotation), *Takifugu* NM_001037985.1 (*TNFA* in the *Takifugu* annotated genome), and *Lepisosteus* XM_015339713.1 (LOC102691380) may correspond in these fish species to the hypothesized TNFSF1/2/3 progenitor. Another TNFSF gene, called “TNF-N” or “TNF-New” (Savan et al. 2005; Glenney and Wiens 2007), is located very close to *Danio TNFB* and *Takifugu TNFA* (Savan et al. 2005). However, they cannot correspond to the hypothesized TNFSF1/2/3 progenitor, because they arose recently; according to the Genomicus database, they are restricted to clupeocephala fishes. Interestingly, the TNF-New sequences are totally different from those of TNFSF1/2/3 genes, suggesting, if they are indeed tandem duplicates of those genes, a very fast divergence following duplication. However, it is possible that they are unrelated to the TNFSF1/2/3 genes and became transposed to a position close to where they are located. In fact, evidence for significant functional differentiation of the TNFSF-New products respect to the TNFSF1/2/3 proteins has been recently obtained (Maeda et al. 2018). The low sequence similarity of TNF-New genes with other TNFs leads to them appearing in different places in the phylogenetic trees (Savan et al. 2005; Glenney and Wiens 2007; Kinoshita et al 2014; Biswas et al. 2015). In our figure 1, they appear at the bottom (see “TNFSF-New group”).

The next orthology group in figure 1 was named TNFSF15. Support for this group is apparently weak, because the bootstrap value for the critical branch is low (just 79%). However, this is due to the inclusion in the group of two rapidly evolving shark sequences (*Callorhinchus* AAVX02013886.1 and *Rhincodon* LVEK02012960.1; see fig. 1). In fact, bootstrap support for the branch that includes the human *TNFSF15/VEGI* gene and two other shark sequences (*Rhincodon* XM_020531692.1 and *Callorhinchus* XM_007909635.1) is very strong (98%). Also, synteny information allows characterizing that most of these genes are located on the same chromosome as human *TNFSFS15/VEGI* (white dots in fig. 1 and supplementary file 2, Supplementary Material online). In addition, very significantly, it was found that the putative *TNFSF15* genes in *Latimeria*, *Lepisosteus*, *Rhincodon* (XM_020531692.1), and *Callorhinchus* (XM_007909635.1) are located in tandem with other TNFSF-encoding genes, included in two strongly supported orthology groups which have been called TNFSF-Fish2 and TNFSF-Fish5 (see their positions in fig. 1 and synteny data in supplementary file 2, Supplementary Material online). This result further reinforces the idea that all the genes included in figure 1 in the TNFSF15 group are orthologous. In humans, *TNFSF8/CD30L* is also located in tandem with *TNFSF15/VEGI* (supplementary file 2, Supplementary Material online), despite the two genes being very different in sequence (fig. 1; see TNFSF8 at the bottom of that figure). A likely *TNFSF8/CD30L* gene was also found in *Latimeria*, (fig. 1), although its exact chromosomal location could not be determined.

The next two groups found in figure 1 are TNFSF3 (already discussed above) and a novel group that has been called TNFSF-Fish1, which includes just a single *Latimeria* and a single *Rhincodon* sequence that appear together in a branch with a highly significant support (98%). Given that there is also strong support (97%) for *TNFSF-Fish1* genes being evolutionarily linked to *TNFSF3* genes and the recent origin of these later, sarcopterygian-specific genes, these results could be interpreted as *TNFSF3* group genes being *TNFSF-Fish1* duplicates, which were subsequently lost in all lineages except sarcopterygians. However, this would be hardly compatible with the existence of the TNFSF1/2/3 tandem; that is, very unlikely events should be postulated to explain why *TNFSF3* appears together with the other two genes. A simpler explanation is that both *TNFSF3/LTβ* and *TNFSF-Fish1* genes are not so closely related, but both being fast-evolving relatives of *TNFSF1/2* and *TNFSF15*, they become separated from those genes and end up together in the tree.

The internal, ancient branch that includes the TNFSF1/2, TNFSF15, TNFSF3, and TNFSF-Fish1 groups has a quite considerable bootstrap value (85%). This association was detected also in other studies, although always, as here, with nonsignificant statistical support (Glenney and Wiens 2007; Biswas et al. 2015). The potential link among all these genes, which from now on will be called as “TNF cluster,” is strongly supported by another kind of information. They are all placed on the MHC complex paralogons emerged in the ancient vertebrate WGDs (Kasahara 1998). Collette et al. (2003) suggested that the regions where TNFSF15 and TNFSF1/2/3 are found derive from the more recent of those two duplications, and all the data obtained in this study are compatible with their hypothesis. In addition of these four groups, the *TNFSF8*, *TNFSF-Fish2*, and *TNFSF-Fish5* genes that, as already mentioned, are found in some species in tandem with *TNFSF15* must, despite their sequence dissimilarity (see locations in fig. 1), also to be included in the TNF cluster, if it is accepted that they are all *TNFSF15* tandem duplicates.

### The EDA Cluster: Early Evolution of EDA, TNFSF13, TNFSF13B, and BALM

A close evolutionary link among three mammalian genes, *EDA*, *TNFSF13/APRIL*, and *TNFSF13B/BLYS* (aka *BAFF*) was pinpointed in several studies (Glenney and Wiens 2007; Premzl 2016; Redmond et al 2017). In addition, a fourth gene called *BALM*, present in some fishes in tandem with *EDA* but absent in mammals, was shown to be also very similar in sequence to those three (Glenney and Wiens 2007; Das et al. 2016; Redmond et al. 2017). These four genes will be grouped in the EDA cluster. EDA-cluster genes are ancient; they were described in chondrichthyans (Pantalacci et al. 2008; Ren et al 2011; Li et al 2012, 2015) and even in lampreys (Das et al. 2016). Collette et al. (2003) proposed that *EDA*, *TNFSF13/APRIL*, and *TNFSF13B/BLYS*, plus a fourth gene that eventually became lost, all emerged from a single precursor gene in the two WGDs occurred in early vertebrate evolution. The duplication that generated the *EDA/BALM* pair would be more recent. In figure 1, these four genes appear together and the branch that includes them all has, despite being ancient, quite a high support (93%). In good agreement with other studies (Glenney and Wiens 2007; Premzl 2016; Redmond et al. 2017), *EDA* is the most divergent of the group, whereas the two most similar are *BALM* and *TNFSF13B/BLYS*. For the EDA and BALM branches, bootstrap support is very high (100% and 99%, respectively), whereas for the TNFSF13/APRIL and TNFSF13B/BLYS groups, it is lower (90% and 83%). However, synteny data are congruent with the tree topology, as shown in figure 1 and supplementary file 2, Supplementary Material online. The four genes are present in sharks, as already discovered by Redmond et al. (2017). In summary, all the available information supports the presence of the four genes of the EDA cluster in the ancestor of the seven species included in figure 1.

The group that appears the closest to the EDA-cluster genes is TNFSF-Fish2 (fig. 1). However, it is very unlikely that this link is real. Not only the bootstrap value of the connecting branch is low (74%) but also, as it has been already indicated, the *TNFSF-Fish2* genes are located in tandem with genes of the TNFSF15/VEGI group. Another gene, *TNFSF12/TWEAK*, is located adjacent to *TNFSF13/APRIL*. The question is whether it is a recent duplicate, perhaps to be included in the EDA cluster. However, both Collette et al. (2003) and Redmond et al. (2017) concluded that the TNFSF12/13 tandem duplication is very ancient. Collette et al. (2003) suggested that it predated the two ancestral vertebrate WGDs. This hypothesis will be explored below, when examining the CD40L cluster.

### The FASL Cluster: TNFSF6 and TNFSF14 Groups

*TNFSF6/FASL* and *TNFSF14/LIGHT* are two genes that have very similar sequences and therefore they appear together in most phylogenetic analyses (see e.g., Collette et al. 2003; Glenney and Wiens 2007; Biswas et al. 2015). We found this association to be strongly supported (bootstrap = 99%; fig. 1). Genes of the TNFSF6 and TNFSF14 groups show a tendency to become duplicated. Multiple *TNFSF6/FASL* has been found in sharks (e.g., four, one of them most likely a pseudogene, are found in tandem in *Callorhinchus*) and two to five *TNFSF14* genes in *Latimeria*, *Danio*, and *Lepisosteus* (fig. 1). From now on, we will call the *TNFSF6/FASL* and *TNFSF14/LIGHT* genes together as “FASL cluster.” Collette et al. (2003) suggested that an ancestral gene located in the MHC region gave rise to the progenitors of the FASL and TNF clusters after becoming duplicated in the first of the early vertebrate WGDs and that *TNFSF6* and *TNFSF14* emerged in the second ancestral WGD. Topology in figure 1 cannot be used to support this hypothesis, given that the FASL cluster appears closer to the EDA cluster than to the TNF cluster. However, the bootstrap value for the critical internal branch is so low (72%) that this putative FASL–EDA link may well be spurious. Thus, our data are not incompatible with Collette et al. (2003) proposal.

### The CD40L Cluster: TNFSF5/10/11/12 and Related Fish Sequences

In figure 1, just below the FASL-cluster genes, six contiguous orthology groups, TNFSF5, TNFSF10, TNFSF11, TNFSF12, TNFSF-Fish3, and TNFSF-Fish4, are defined. They will be called the “CD40L cluster,” following the name of one of the most analyzed human genes, *TNFSF5/CD40L*. They are included together in an ancient branch, although with very low bootstrap value (67%). All of these groups have maximum bootstrap support (100%) except TNFSF5, which is however strongly supported by synteny data (black dots in fig. 1 and see details in supplementary file 2, Supplementary Material online). Several studies already pinpointed the close similarity between *TNFSF10* and *TNFSF11*, and, in some cases, their relationships with *TNFSF5*, as well as the presence in fishes of additional, closely related genes (Collette et al. 2003; Glenney and Wiens 2007; Biswas et al. 2015; Premzl 2016). However, no previous phylogenetic tree showed that those genes are related to *TNFSF12*, which indeed has a quite different sequence (see the long branch for *TNFSF12* genes in fig. 1). However, notably, Collette et al. (2003) suggested that mammalian *TNFSF5*, *-10*, *-11*, and *-12* all derived from a single ancestral gene, quadruplicated in the two early rounds of WGD in vertebrates. Given that the four genes are already present in sharks and analyses in figure 1 demonstrate that they have quite similar sequences, Collette et al. (2003) hypothesis receives here a significant endorsement. In addition, synteny analyses suggested that both *TNFSF-Fish3* and *TNFSF-Fish4* genes may be *TNFSF5* duplicates, because in the three cases the human orthologs of the genes that surround them are found on the X chromosome (supplementary file 2, Supplementary Material online). Actually, *TNFSF-Fish4* genes are detected in marsupials and monotremes (e.g., wombat LOC114053674; platypus LOC107547878), in exactly the same position that in fishes, meaning that this gene has been lost in the eutherian lineage. Except for *TNFSF-Fish3*, which has been lost several times, only isolated species-specific duplications and losses of CD40-cluster genes are observed (fig. 1).

### The 4-1BBL Cluster: TNFSF4/7/9/18; Odds and Ends: Highly Divergent Sequences

A large ensemble of heterogeneous sequences that cannot be ascribed to any of the four clusters hitherto defined appear together at the bottom of figure 1. Among them, the five highly divergent sequences, already mentioned at the beginning of this section, which could not be included in any orthology group, are found (see asterisks in fig. 1). In addition, seven orthology groups (TNFSF-Fish5, TNFSF9, TNFSF4, TNFSF18, TNFSF-New, TNFSF7, and TNFSF8) are also located in this region of the tree. The five groups that include two or more sequences all have strong bootstrap support (95–100%) except for TNFSF-New, but the orthology of the TNFSF-New sequences detected in *Danio* and *Takifugu* is well supported by synteny data, as already described above (see TNF Cluster section).

The internal branch of the tree that contains all these sequences has a very low bootstrap support (65%). Thus, whether these genes are indeed related or they just appear artificially together in our ML analyses is, at first sight, unclear. However, additional evidence supports that some of them are indeed evolutionary linked. A first significant information is that human *TNFSF7/CD70* and *TNFSF9/4-1BBL* are located in tandem on chromosome 19. According to data in the Genomicus database, *TNFSF7/CD70* is mammalian-specific, and thus can be interpreted as a recent duplicate of *TNFSF9/4-1BBL*. This explains why no ortholog was found in fishes in our searches. Similarly, *TNFSF4/OX40L* and *TNFSF18/AITRL* are located in humans in a tandem on chromosome 1. Again, *TNFSF18/AITRL* is, according to Genomicus, also a recent, mammalian-specific duplicate, and thus it was of course impossible to detect in the fishes examined here. Evidence exists for these two tandems having a deep evolutionary relationship, because in both of them an additional gene of the FASL cluster is found. For the *TNFSF8/14* couple, the third gene in the tandem is *TNFSF6/FASL*, whereas for the *TNFSF7/9* pair, that third gene is *TNFSF14/LIGHT* (details in supplementary file 2, Supplementary Material online). Given the strong similarity of *TNFSF6/FASL* and *TNFSF14/LIGHT* that we have already discussed (see FASL Cluster section), the simplest explanation is that both tandems have a common evolutionary origin. Thus, a logical hypothesis is that, before the last WGD occurred in early vertebrate evolution, there were already two ancestral genes located in tandem. One of them would be the progenitor of *TNFSF6/FASL* and *TNFSF14/LIGHT* and the second one the progenitor of both *TNFSF9/4-1BBL* and *TNFSF4/OX40L*. Then, the second WGD produced the two distinct tandems that we now observe. This model is in good agreement with the results shown in figure 1. Notice also that, again supporting an ancient origin, *TNFSF6/FASL*, *TNFSF14/LIGHT*, *TNFSF9/4-1BBL*, and *TNFSF4/OX40L* are all present in sharks. The evolutionary ensemble of the ancient genes *TNFSF4/OX40L* and *TNFSF9/4-1BBL* and their two recent duplicates *TNFSF7/CD70* and *TNFSF18/AITR* will be called from now on the 4-1BBL cluster.

We are left with three groups, TNFSF8, TNFSF-Fish5, and TNFSF-New, which appear close to 4-1BBL-cluster genes in figure 1, although it is clear that they are not related to genes in that cluster, being either *TNSF15* or *TNFSF1/2* duplicates (see above, TNF Cluster section). Collette et al. (2003) interpreted the *TNFSF8/TNFSF15* tandem to be ancient, existing before the ancestral vertebrate WGDs. However, *TNFSF8/CD30L* is not observed in fishes other than *Latimeria*.

### TNF Superfamily Genes in Agnathans

**Fig. 2:**
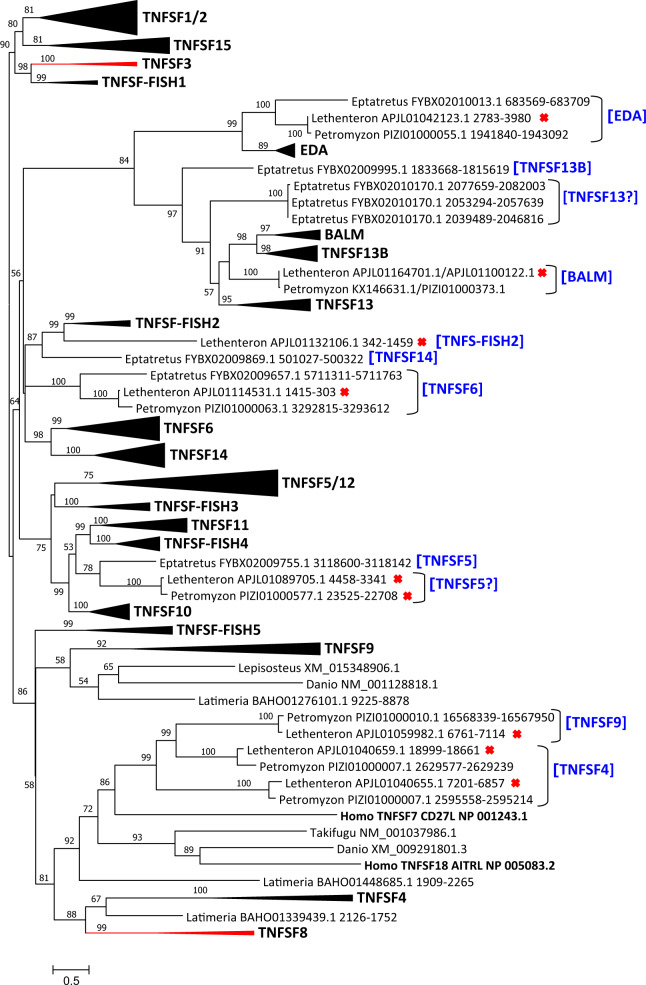
ML tree including both agnathan and gnathostome TNFSF sequences. Bootstrap values and names of sequences are indicated as in figure 1. The gnathostome orthology groups described in figure 1 have been here collapsed (triangles). Orthology groups that include shark sequences are shown in black color. Red triangles refer to groups in which shark sequences are absent. The orthology groups deduced for the agnathan sequences, based on sequence similarity and synteny, are shown in brackets (blue color). For five agnathan sequences, information is insufficient to ascribe them to any orthology group. Given where they are placed on the three, they may correspond to *TNFSF13* and *TNFSF5* orthologs, but a question mark has been added to indicate that such assignment still lacks support. In table 1, these five sequences are included in the “Other” class, with all the sequences not ascribed to any orthology group.

So far, only a few TNFSF genes had been described in agnathans. Suzuki et al. (2004) found two in the hagfish *E. burgeri*, which they considered, based on sequence similarity, as potential orthologs of, respectively, *TNFSF10/TRAIL* and *TNFSF13/APRIL*. On the other hand, Das et al. (2016) characterized a gene similar to *TNFSF13B/BLYS* (aka *BAFF*) present in the lampreys *P. marinus* and *Let. camtschaticum*. This gene was interpreted as equally similar, co-orthologous, to *TNFSF13B/BLYS* and *BALM* by Redmond et al. (2017). Given that, in the previous sections, we are hypothesizing an ancient origin of many TNFSF genes, in several cases originating before the ancestral vertebrate WGDs, it would be unexpected to find very few genes in agnathans. Indeed, exhaustive searches characterized a relatively large set of TNF-like sequences both in lampreys and in hagfishes. Their phylogenetic relationships with the orthology groups defined for the rest of vertebrates are summarized in figure 2. The corresponding alignment can be found in supplementary file 3, Supplementary Material online. This phylogenetic tree demonstrates that, as expected, very different TNF superfamily members exist in agnathan species. It is significant that the sets of TNFSF sequences found in the lampreys *Petromyzon* and *Lethenteron* are almost identical and very similar to those found in the hagfish *Eptatretus*. The agnathan sequences are found in eight different places in the tree, with likely orthologous genes being found in two or the three species in seven out of these eight cases. Thus, it is very unlikely that many more TNFSF genes are to be found in these species; if this was the case, many agnathan sequences should appear isolated. This conclusion is in good agreement with an estimation of genome completeness for *Petromyzon*, which established that practically all single-copy vertebrate genes expected to be found in that lamprey are indeed detected (Smith et al. 2018).

No agnathan sequences are similar to those of the TNF-cluster genes *TNFSF1/2*, *TNSF3*, *TNFSF15*, or *TNFSF-Fish1* (fig. 2, top). It was postulated above that two ancient progenitors of, respectively, the *TNSF15/TNFSF-Fish2/TNFSF-Fish5/TNFSF8* genes and the *TNFSF1/TNFSF2/TNFSF3* genes existed after the two WGDs that occurred before the agnathan/gnathostome split. The lack of obvious *TNFSF1/2/3* and *TNFSF15* genes in agnathans can be interpreted as indicating that these two progenitors were lost in the agnathan lineage, although it cannot be ruled out that these genes are still to be found in these incompletely assembled genomes. Most interestingly, a *Lethenteron* gene is found in a highly supported branch (99% bootstrap value) with *TNFSF-Fish2* genes. Given that *TNFSF-Fish2* is a tandem duplicate of *TNFSF15*, this result suggests the existence of not two but three ancient TNF-cluster genes before the agnathan/gnathostome split. It also indicates that the strong *TNFSF15* versus *TNFSF-Fish2* sequence divergence that is now detected occurred very early in vertebrate evolution.

Five similar sequences in *Eptatretus* and two in both *Petromyzon* and *Lethenteron* appear very close in the tree to gnathostome EDA-cluster genes (fig. 2). A gene for each agnathan species appears very close to *EDA* itself, in a highly supported branch (bootstrap = 99%), The other six sequences appear in a second branch, also highly supported (97%), which includes the other three EDA-cluster genes, *TNFSF13*, *TNFSF13B*, and *BALM* (fig. 2). This indicates that at least two EDA-cluster genes were already present before the agnathan/gnathostome split. However, data described above (see EDA Cluster section) suggested that not two but the four EDA-cluster genes already existed after the two rounds of WGD that preceded the agnathan/gnathostome split, Indeed, synteny data suggest that *Petromyzon* KX146631.1/PIZI010100373.1 most likely is a *BALM* ortholog, given that it is adjacent in *Petromyzon* to *IGBP1*, a gene that is found very close to the EDA/BALM tandem in other species (see details in supplementary file 2, Supplementary Material online). Also, adjacent to *Eptatretus* FYBX02009995.1 is found *ABHD13*, which is in exactly the same position respect to *TNFSF13B* in other species (supplementary file 2, Supplementary Material online). Finally, it was impossible to determine from which region of the genome come the TNFSF sequences found in *Eptatretus* FYBX02010170.1. They may be additional, *Eptatretus*-specific duplicates, although it is also possible that they correspond to the fourth EDA cluster gene, *TNFSF13* (fig. 2).

It was established above that both genes of the FASL cluster, *TNFSF6* and *TNFSF14*, existed prior to the agnathan/gnathostome split. A single sequence of each of the three agnathan species analyzed appear in a highly supported branch (bootstrap = 100%) relatively close to those two orthology groups. Synteny data suggest that these sequences may correspond to *TNFSF6* orthologs, given that the genes that are closest to them in the agnathan genomes have human orthologs on chromosome 1, precisely where *TNFSF6* is located (supplementary file 2, Supplementary Material online). In addition, *Eptatretus* FYBX02009869.1 is surrounded by genes whose orthologs are in human chromosome 19p13.2–p13.3 (supplementary file 2, Supplementary Material online). In figure 2, it can be observed that *Eptatretus* FYBX02009869.1, although quite similar to *TNFSF-Fish2* genes, is actually in an intermediate position between those genes and the genes of the FASL cluster. Given that *TNFSF14* is located in humans precisely on chromosome 19p13.3, it is likely that the *Eptatretus* FYBX02009869.1 sequence corresponds to a divergent *TNFSF14* ortholog.

Once again, a single gene in each of the three agnathan species appears in a highly supported branch (bootstrap = 99%) with the gene groups TNFSF10, TNFSF11, and TNFSF-Fish4, which belong to the CD40L cluster (fig. 2). A minimum of four genes of this cluster, *TNFSF10*, *TNFSF11*, *TNFSF12*, and the *TNFSF5/TNFSF-Fish3/TNFSF-Fish4* progenitor were postulated to exist after the ancestral vertebrate WGDs (see CD40L section above). This result suggests that three CD40L-cluster genes have been lost in agnathans. When synteny was analyzed, it was found that the genes adjacent to the TNFSF genes in *Eptatretus* FYBX02009755.1 (only sequence for which synteny can be ascertained) have orthologs located on human chromosome X, just as happens for *TNFSF5*, *TNFSF-Fish3*, and *TNFSF-Fish4*. A particular gene, *RBMX*, is very close to the TNFSF gene in FYBX02009755.1 and to *TNFSF5* in other species (supplementary file 2, Supplementary Material online), suggesting that the agnathan gene is a true *TNFSF5* ortholog. A rapid evolution of the gnathostome *TNFSF5* genes may explain why this agnathan gene is more similar to the other CD40L-cluster genes than to the *TNFSF5* genes themselves. The other two agnathan CD40L-like sequences (*Lethenteron* APJL01089705.1 and *Petromyzon* PIZI01000577.1) may also correspond to *TNFSF5* orthologs, but given that the bootstrap support for their connection with *Eptatretus* FYBX02009755.1 is low, other possibilities remain open.

Finally, a group of three very similar sequences was detected in both *Petromyzon* and *Lethenteron* (fig. 2, bottom). Synteny was determined for the *Petromyzon* sequences (information available for the *Lethenteron* ones was insufficient). Two of them, located in tandem on contig PIZI0100007.1, are surrounded by genes whose human orthologs are present on chromosome 1 and the third, PIZI01000010.1, is close to genes whose orthologs are in human chromosome 19. Thus, in spite of their very limited sequence similarity, these agnathan sequences may correspond to, respectively, *TNFSF4* (located in humans on chromosome 1) and *TNFSF9* (in our species, located on chromosome 19). Precisely, these two genes were deduced to be ancient, existing already after the early vertebrate WGDs (see above, 4-1BBL section).

### A Model for TNF Superfamily Evolution in Early Vertebrates

Table 1 summarizes the results obtained for the nine fish species plus humans. It can be observed that all the analyzed gnathostome species have a comparable level of complexity, with a number of genes ranging from 14 (*Takifugu*) to 28 (*Rhincodon*) and a number of orthology groups present that ranges between 13 (again *Takifugu*) and 19 (*Latimeria*), out of a maximum of 24. The difference in the number of genes is in part due to tandem duplications that occurred more often in some species than in others. However, some reductions in the number of genes, involving multiple losses, can also be deduced when closely related species are compared (e.g., *Takifugu* vs. *Danio*; *Homo* vs. *Latimeria*). In agnathans, both the number of genes (8–9) and the number of orthology groups present (5–6) are much smaller.

**Table 1:** Number of Genes Included in Each Orthology Group in the Ten Species Analyzed in This Study

Species	Orthology Groups																									
	1/2	3	4	5	6	7	8	9	10	11	12	13	13B	14	15	18	EDA	BALM	NEW	F1	F2	F3	F4	F5	Other	No.
Petromyzon marinus			2		1			1									1	1							1	7
Lethenteron camtschaticum			2		1			1									1	1			1				1	8
Eptatretus burgeri				1	1								1	1			1								3	8
Callorhinchus milii			1	1	4			1	1	1	1	1	1		2		1	1			1	1	1	1		20
Rhincodon typus	8		1		3			1	1	1	1	1	2	1	2		1	1		1	1	1	1	1		29
Danio rerio	2			1	1			1	1	1	1	1	1	2	1		1		1			1	2		1	19
Takifugu rubripes	1			1	1			1	2	1	1	1		1			1	1	1				1			14
Lepisosteus oculatus	1			1	1				1	1	1	1	1	5	1		1	1				1	1	1	1	20
Latimeria chalumnae	3	1		1	1		1	1	1	1	1	1	1	2	1		1	1		1	1		1	1	3	25
Homo sapiens	2	1	1	1	1	1	1	1	1	1	1	1	1	1	1	1	1									18

Note.—Other, genes not included in any orthology group; No., total number of genes.

**Fig. 3:**
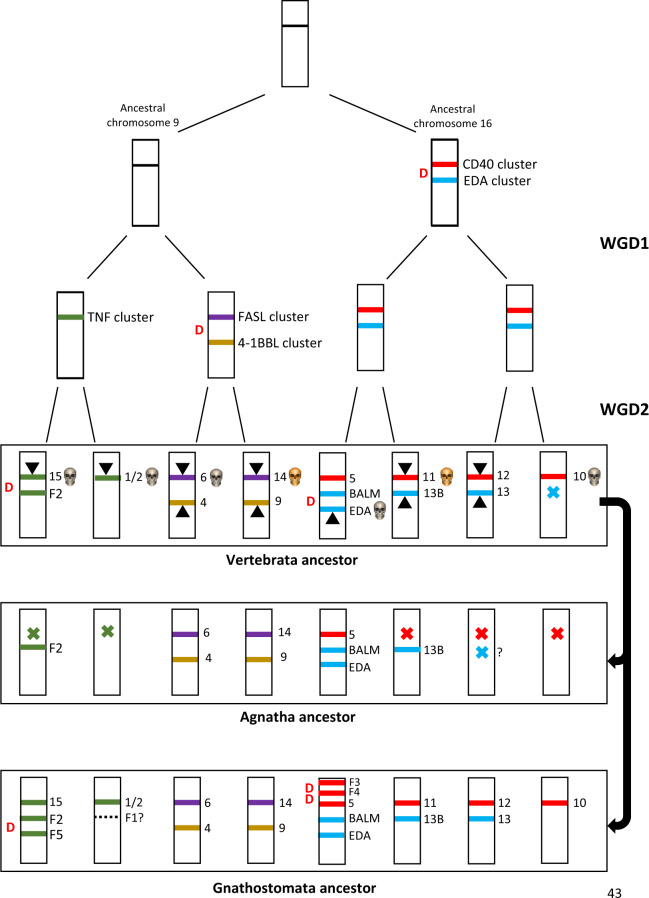
Model for the early evolution of the TNF superfamily. The origins of the progenitor genes of the CD40, EDA, TNF, FASL, and 4-1BBL clusters are indicated. All genes of a given cluster are depicted with the same color. WGD1 and WGD2 are the two rounds of genome duplication occurred in early vertebrate evolution, before the agnathan/gnathostome split (see text). D indicates a tandem duplication event. Gene losses are indicated with crosses. In the box that corresponds to the vertebrate ancestor, genes that encode proteins that in modern mammals are known to interact with death-domain-containing receptors are marked with gray skulls. A golden skull means that the gene encodes a ligand that interacts with either TNFSF6B or TNFSF11B, that, despite not acting today as death receptors, still retain in their sequences similarity to death domains, meaning that intracellular death-domain-dependent cascades could be activated by those receptors in the past. Triangles indicate genes whose products activate, when interacting with the corresponding receptors, the NF-κB pathway.

**Fig. 4:**
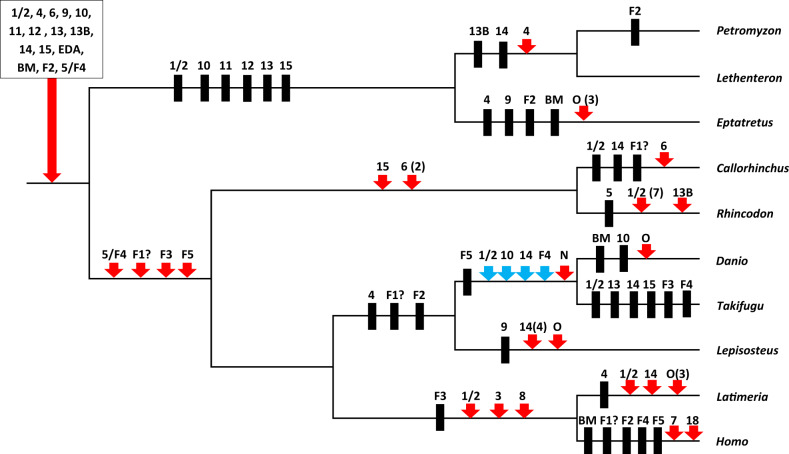
Evolutionary history of the TNFSF genes along vertebrate evolution. Fifteen genes are deduced to exist prior to the divergence of all these species (box). Black rectangles indicate gene losses and red arrows, gene duplications. Blue arrows indicate the genes that emerged in the teleost-specific WGD discussed in the text and are still found in either *Danio* or *Takifugu*. Numbers in parentheses refer to the number of duplicates; lack of number indicates a single duplicate. Question marks refer to the uncertainties to trace the evolutionary history of TNFSF-Fish 1. O, “other,” that is, genes not included in any orthology group; F, Fish; BM, BALM; N, New.

With the information described in the previous sections, it is possible to propose a hypothesis of how the TNF superfamily evolved in early vertebrates. We will follow the steps of Collette et al. (2003), which combined all the information then available to generate the simplest model to fit their data. Figure 3 shows the most parsimonious explanation to accommodate all the available data obtained here. It starts assuming a single TNF superfamily gene, present in an invertebrate ancestor of modern vertebrates, and indicates: 1) the most likely time of emergence of the progenitors of the five gene clusters defined in this work (i.e., TNF, EDA, CD40, FASL, and 4-1BBL); 2) the impact of the two WGDs; and 3) the diversity of the TNF superfamily just after those WGDs and also later, in the ancestors of agnathans and gnathostomes. Figure 4 indicates the patterns of emergence of loss of each gene in the ten lineages considered in this study. If we compare figure 3 and the hypothesis developed by Collette et al. (2003), it is obvious that there are significant similarities. The main one is that many genes emerged as a consequence of the two WGDs. TNFSF genes located before these duplications in the ancestral chromosomes 9 and 16 (according to the nomenclature developed by Sacerdot et al. [2018]) became, as first suggested by Collette et al. (2003), distributed by their effects in eight different chromosomes. As already indicated, in humans, they are, on one hand, chromosomes 1, 6, 9, and 19 (corresponding to the MHC paralogons; Kasahara 1998) and, on the other hand, chromosomes 3, 13, 17, and X. Genes of the TNF, FASL, and 4-1BBL clusters are distributed in the first quartet of chromosomes, whereas genes of the CD40 and EDA clusters are found in the second quartet (fig. 3). However, despite this general similarity, if we carefully compare the model proposed by Collette et al. (2003) and the one summarized in figure 3, it becomes obvious that the details are very different. Particularly, the hypothesis presented here is much simpler. Collette et al. (2003) postulated the emergence of six TNFSF genes before the ancestral WGDs, with two of them being lost just before them. On the contrary, here it is hypothesized the presence of just three genes before the WGDs, and no losses are required. Similarly, Collette et al. (2003) suggested that 15 TNFSF genes were present after the WGDs, whereas only 13 are hypothesized here (fig. 3). If we consider that they missed in their analyses the seven fish-specific genes (*TNFSF-Fish1* to *TNFSF-Fish5*, *BALM*, and *TNFSF-New*) detected in this and other studies, we can conclude that their hypothesis necessarily would have become even more complex to accommodate the whole data set analyzed here. These differences are mainly due to Collette et al. (2003) suggesting the very early presence, much before the WGDs, of a tandem of three genes, being one of them the progenitor of *TNFSF3*, *-4*, *-7*, *-8*, *-9*, and *-18*. However, it has been demonstrated in previous sections that, of all those, only *TNFSF4* and *TNFSF9* are present in sharks and most likely in agnathans (figs. 1 and 2 and text above), whereas the rest emerged much more recently. Thus, there is no objective evidence for the existence of such a progenitor gene so early in vertebrate evolution. We propose the much simpler hypothesis that the progenitor of all the 4-1BBL-cluster genes (*TNFSF4*, *-7*, *-9*, and *-18*) emerged just after the first WGD (fig. 3). We also showed that *TNFSF3*, *TNFSF8*, and *TNFSF18* are still much more recent, being present only in sarcopterygians (figs. 1 and 3). Their evolutionary histories are thus totally different from the ones assumed by Collette et al. (2003).

Some independent evidence supporting our hypothesis of just three ancestral genes before the vertebrate WGDs can be obtained from the analysis of the TNFSF genes present in the cephalochordate *Branchiostoma floridae* performed by Huang et al. (2008). Although their phylogenetic analyses comparing *Branchiostoma* and vertebrate genes are far from conclusive (e.g., they did not included the whole set of TNFSF vertebrate genes; bootstrap support for the topology obtained is weak), they indeed suggested that *Branchiostoma* TNFSF genes can be divided into four groups, which can be easily explained as derived from our three ancestral genes. The first set includes genes quite similar to vertebrate *TNFSF10* and *TNFSF11* (both of the CD40 Cluster, fig. 3); the second, they consider similar to *TNFSF2* and *TNFSF6*, which belong to, respectively, the TNF and FASL clusters, both derived from the progenitor gene in ancestral chromosome 6 (see again fig. 3); the third, they found was quite similar to *TNFSF5* (again CD40 cluster); finally, the fourth set of genes was most similar to both *TNFSF13* and *EDA* (both members of the EDA cluster, fig. 3). These results suggest that the three original genes postulated in our model were already present before the split that separated the cephalochordate and vertebrate lineages. In this context, additional significant results were found by Robertson et al. (2006) in the echinoderm *Strongylocentrotus purpuratus*. They described four TNFSF genes in that species, two of them most similar to *EDA* and the other two to *TNFSF1/-2/-3*. This suggests that the first duplication postulated on top of figure 3 may have occurred already before the echinoderm/chordate split. However, again, the analysis by Robertson et al. (2006) is very incomplete and the topology that they obtained is poorly supported. We must therefore conclude that the model shown in figure 3 is compatible with all the available evidence, but more precise analyses comparing vertebrate and invertebrate TNFSF genes are required to rigorously test whether it indeed fully explains the early evolution of this gene family as well as to determine the precise timing of the earliest TNFSF duplications.

From the summary shown in figure 4, in which the patterns of duplication and loss are detailed, it can be deduced that the common ancestor of agnathans and gnathostomes already had a complex TNFSF/TNFRSF system, with about 15 genes. After the agnathan/gnathostome split, both lineages followed opposite trends: although a large simplification of the system occurred in agnathans, a significant increase occurred in gnathostomes. More recently, varied and complex remodelings of the TNFSF/TNFRSF system, with emergence of new genes plus a substantial number of gene losses, are observed, with a tendency of additional losses in agnathans and to stabilization or some further increase in the number of TNFSF genes in gnathostomes. It is however noteworthy the decrease observed in our own lineage, with five relatively recent gene losses (fig. 4). Actually, it is deduced that we have one TNFSF gene less than the ancestor of all gnathostomes, which lived about 475 Ma (Betancur-R et al. 2017; Irisarri et al. 2017). It is also interesting that the teleost-specific WGD failed to determine a significant increase in the number of TNFSF genes. Although some increase related to that WGD is observed in *D. rerio*, *T. rubripes* has fewer genes than the ancestor that suffered that duplication (fig. 4). This means that practically all genes emerged in that WGD became lost. This is the opposite of what occurred in early vertebrate evolution, in which practically all the genes derived from the two WGDs persisted (fig. 3).

### TNFSFs in Early Vertebrates: Functional Implications of the Evolutionary Patterns

Assuming that the evolutionary hypothesis summarized in figure 3 is correct and that TNFSF proteins have retained similar functions since they emerged, several significant functional implications can be inferred. First, from the three original TNFSF genes that existed before the vertebrate WGDs, multiple descendent genes encode proteins that interact with receptors of the TNFR superfamily that contain death domains (see details in fig. 3). Thus, it can be inferred that TNFSF ligands were involved in cell-death regulation through activation of death-domain-containing receptors (extrinsic apoptotic pathway; Legrand et al. 2019) already very early in evolution, probably before vertebrates emerged. This is in perfect agreement with the finding of TNFSF receptors with death domains in invertebrates and nonvertebrate chordates (Quistad and Traylor-Knowles 2016) and the observations that human TNFSF2/TNF protein is able to induce apoptosis in corals and also that TNF-like proteins encoded in corals induce apoptosis in human cells through FADD, a protein required for the activation of caspases by death domain TNFRs (Quistad et al. 2014). Similarly, it can be deduced that TNFSF-dependent activation of the canonical and/or noncanonical NF-κB pathways, critical for inflammation and to respond to pathogens (Zhang et al. 2017), is a feature that probably arose very early, given that, again, genes derived from all three pre-WGDs TNFSF progenitor genes encode proteins that are involved in signaling that leads to activation of one or both of those pathways (see also details in fig. 3).

It is also interesting to divide the TNFSF genes into three groups: 1) those that emerged early and have been retained in most/all vertebrates, including both agnathans and gnathostomes; 2) those that emerged early but were lost in agnathans; and 3) those that emerged more recently and are found only in gnathostomes. According to data summarized in table 1, there are just two strictly conserved genes, found in all species analyzed, *TNFSF6/FASL* and *EDA*. Similarity of multiple invertebrate TNFSF genes with *EDA* has been described (Wiens and Glenney 2011). Six other genes, namely *TNFSF4*, *-9*, *-13B*, *-14*, *BALM*, and *TNFSF-Fish2*, although lost in one or several of the species analyzed, have been found in both agnathans and gnathostomes (figs. 3 and 4 and table 1). It is significant that this conserved group includes genes of all five ancestral clusters, TNF, 4-1BBL, FASL, CD40, and EDA. This may be interpreted as indicating a very early division of labor among genes belonging to different clusters, in such a way that eliminating all genes of a cluster became very difficult. Only *Eptatretus* apparently lacks both TNF-cluster and 4-1BBL-cluster genes, whereas in *Petromyzon* (but not in its close relative *Lethenteron*) no TNF-cluster genes have been detected. Whether this indeed means that these jawless fishes have found a way to survive without those kinds of genes or it simply indicates that one/two additional TNFSF genes have to be found in their genomes remains to be elucidated.

No less than six genes present in the common ancestor of agnathans and gnathostomes (*TNFSF1/2*, *-10*, *-11*, *-12*, *-13*, and *-15*) have not been detected in jawless fishes. Three of them (*TNFSF1/2*, *-10*, and *-15*) encode ligands that in mammals interact with death-domain-containing TNFRs. Moreover, TNFSF11 protein interacts with TNFRSF11B, a decoy receptor that, despite not being a membrane protein, also has two regions with significant similarity with death domains (fig. 3; Yamaguchi et al. 1998), suggesting that its original function may also have been the regulation of apoptosis. It is interesting that the opposite is found if we consider the five genes that emerged after the agnathan/gnathostome split. All of them (*TNFSF3*, *-5*, *-7*, *-8*, and *-18*) encode proteins that in mammals bind TNFRs without death domains (Dostert et al. 2019). It is true that TNFSF3/LTβ protein forms heterotrimers with TNFSF1/LTα (Browning et al. 1993) that then are able to bind to the death-domain-containing receptor TNFRSF1A/TNFR1. However, because TNFSF1/LTα is usually secreted as a homotrimer and TNFSF3/LTβ also forms homotrimers that remain membrane-bound and then interact with a different receptor, TNFRSF3/LTβR, which lacks a death domain (Dostert et al. 2019), their combined action as membrane-bound heterotrimers may be interpreted as a secondary role for both the TNFSF1/LTα and the TNFSF3/LTβ products, perhaps a vestige of the roles that fulfilled the proteins generated by the progenitor of both genes, which has persisted due to the similarity that both proteins still retain.

## Discussion

The main goal of this study was to determine the patterns of emergence, diversification and, potentially, simplification of the TNF superfamily in early vertebrate evolution. A set of fish species was carefully chosen as the best models for that kind of analysis. It was self-evident the need to include early-diverging lineages. This is the first work in which the TNFSF genes of hagfishes, lampreys, and sharks are studied in detail. The model bony fishes *D. rerio* and *T. rubripes* were also included because their TNFSF genes had been already carefully studied and compared with those in mammals (Glenney and Wiens 2007; Biswas et al. 2015). We reasoned that they could provide significant clues to understand the results for the other species. Two additional bony fishes, the spotted gar *Lep. oculatus* and the coelacanth *L. chalumnae*, were included for the additional advantages that they provided. Genes of the holostean *Lepisosteus* have been shown to evolve slowly respect to those of teleosts (Braasch et al. 2016). Moreover, *Lepisosteus* diverged from *Danio* and *Takifugu* about 300 Ma, before an additional, teleost-specific WGD occurred (Near et al. 2012; Braasch et al. 2016; Ravi and Venkatesh 2018; Hughes et al. 2018). Finally, *Latimeria* is a sarcopterygian, a member of the evolutionary branch from which our own species emerged. It was included as a useful link between mammals and the other, more distantly related, fishes.

By performing exhaustive database searches, all the TNFSF genes of these species have been discovered and classified (figs. 1–4 and table 1). As detailed above, a substantial number of new genes have been found in the fish species whose TNF superfamily had been already analyzed (*Danio*, *Takifugu*, *Latimeria*, and *Callorhinchus*). The addition of the TNFSF genes present in the other five fish species provides a much more precise view of TNFSF diversity and provides the first complete data set from which general conclusions on the evolution of this superfamily in early vertebrates can be obtained. Phylogenetic analyses combined with synteny data have allowed classifying TNFSF genes into 24 orthology groups. Seventeen of them have been named according to the human gene (or genes, as in the TNFSF1/2 group) that they include. The other seven groups do not incorporate a human ortholog. In two cases (BALM and TNFSF-New), they had been already characterized by other authors (Savan et al. 2005; Glenney and Wiens 2007; Das et al. 2016; Redmond et al. 2017). The other five fish-specific orthology groups (TNFSF-Fish1 to -Fish5) are described here for the first time.

By analyzing the whole data set, it was possible to characterize the most likely time when each of the genes emerged (figs. 3 and 4). This led to the definition of five TNF superfamily clusters, groups of evolutionary related genes, which derive from five specific progenitors already present before the agnatha/gnathostomata split. The functional significance of these clusters is clear. The proteins derived from genes included in a given cluster often show overlapping interactions with particular receptors of the TNFR superfamily (summarized by Dostert et al. [2019]). Thus, the products of the TNF-cluster genes TNFSF1/LTα and TNFSF2/TNF both interact with the TNFRSF1A/TNFR1 and TNFRSF1B/TNFR2 receptors; the EDA-cluster genes TNFSF13/APRIL and TNFSF13B/BLYS (aka BAFF) generate ligands able to interact with receptors TNFRSF17/BCMA and TNFRSF13B/TACI; the products of the FASL-cluster genes TNFSF6/FASL and TNFSF14/LIGHT both interact with TNFRSF6B/DCR3; and, finally, the products generated from TNFSF10/TRAIL and TNFSF11/RANKL both interact with receptor TNFRSF11B/OPG. If we consider (fig. 1) that three of these pairs of TNFSF genes existed already before the chondrichthyan/osteichthyan split (about 450–475 Ma; Betancur-R et al. 2017; Irisarri et al. 2017) and the most recent pair (*TNFSF1/LTα* and *TNFSF2/TNF*) was already present before the split that separated the coelacanth and human lineages (about 425 Ma; Betancur-R et al. 2017; Irisarri et al. 2017), this functional conservation is striking. It is also significant that only two TNF receptors (TNFRSF3/LTβR and TNFRSF6B/DCR3) are able to interact with proteins encoded by TNFSF genes of different clusters (Dostert et al. 2019). Another striking demonstration of functional similarity among the products of genes of the same cluster is the fact that heterotrimers are generated in vivo between TNFSF1/LTα and TNFSF3/LTβ proteins and also between TNFSF13/APRIL and TNFSF13B/BLYS proteins (Browning et al. 1993; Roschke et al. 2002).

The patterns described for the evolution of the TNF superfamily (figs. 3 and 4) suggest that it has expanded due to two different processes. First, as a consequence of the early vertebrate WGDs, which, from just three progenitors, generated a set of about 15 genes (fig. 3). Almost all the genes derived from these WGDs have survived in gnathostomes, whereas many have been lost in agnathans (figs. 3 and 4). Second, by relatively frequent gene duplications that generated tandems of similar genes. Several examples of these tandem duplication events were already described in the previous sections. On the contrary, most of the genes emerged in the more recent WGD occurred in the teleost lineage have later disappeared (fig. 4). It is tempting to speculate that the early expansion of the TNF superfamily may have allowed its members to become key actors in the development and regulation of the adaptive immune system in early gnathostome evolution, with the other, more recently duplicated genes contributing additional roles that allowed further refinement of that system. Such an acquisition of novel roles may be much more difficult once the immune system was fully established, explaining why most additional TNFSF genes generated by the teleost WGD became dispensable and were lost. In agnathans, which have an alternative adaptive immune system (Boehm et al. 2018; Flajnik 2018), the TNF superfamily has become substantially simplified. It is possible that the establishment of this alternative immunity required a simpler pattern of cell–cell interactions mediated by the TNFSF/TNFRSF system, so gene losses were easily accommodated.

It has been deduced from the patterns of emergence of TNFSF genes and their current roles in mammals that both their involvement in the regulation of cell death and their roles in NF-κB pathway control are ancient. There is direct evidence for the control of apoptosis by TNFSFs acting in invertebrate animals, such as cnidarians (Quistad et al. 2014). However, so far there is no evidence for TNFSF proteins being able to activate the NF-κB pathway in invertebrate species. This pathway, present in all animals including sponges (Riesgo et al. 2014), most likely emerged before metazoans split from their closest protozoan relatives, given that both choanoflagellates and the ichthyosporean *Capsaspora* contains genes that encode proteins very similar to those involved in the pathway in animals, including NF-κB itself (Gilmore and Wolenski 2012; Richter et al. 2018). However, when the intracellular effects of the TNFSF/TNFRSF system have been analyzed in invertebrates, a role in NF-κB control has not been found. In particular, the function of the only TNFSF gene of *Drosophila melanogaster*, *Eiger*, has been extensively studied. Signaling through the TNFSF receptors Wengen and Grindelwald does not activate the NF-κB pathway, but a different one, JNK (Moreno et al. 2002; Kauppila et al. 2003; Andersen et al. 2015). Overexpression of a TNFRSF gene of the crustacean *Litopenaeus vannamei* in *Drosophila* cells also failed to activate their NF-κB pathway (Wang et al. 2012). This may indicate that the control of the NF-κB pathway by the TNFSF/TNFRSF signaling system is absent in invertebrates. It has been suggested that the NF-κB pathway was originally controlled by signals derived from Toll-like receptors as part of the innate immune response (Gilmore and Wolenski 2012) and only much more recently coopted by the TNFSF/TNFRSF system to contribute to adaptive immunity (Moreno et al. 2002; Collette et al. 2003; Wiens and Glenney 2011). Analyzing the functions of TNFSFs genes in invertebrates other than *Drosophila* or in nonvertebrate chordates may contribute to determine the precise moment in which that switch occurred.

Finding that several TNFSF genes lost in agnathans have in mammals roles in the extrinsic apoptotic pathway but none of the genes emerged in gnathostomes has main roles in that pathway may be a very significant clue to understand the different functions of the TNFSF/TNFRSF system in the two vertebrate branches. The fact that jawless fishes have less TNFSFs able to interact with TNFRs with death domains suggest that, relative to early vertebrates or modern gnathostomes, the activation of cell-death mechanisms by TNFSFs is temporally restricted along development and/or involves a more limited set of cell types in agnathans. On the other hand, the discovery that the gnathostome-specific TNFSFs have receptors that do not have death domains indicates that these duplicates emerged to refine cell-to-cell interactions, that is, to generate additional, specific ways to induce the activation of intracellular regulatory cascades (Borst et al. 2005; Elgueta et al. 2009; Nocentini and Riccardi 2009; van der Weyden et al. 2017). In summary, it can be inferred that, since their split from the agnathan branch, gnathostomes have evolved a more sophisticated TNFSF-based system of cell communication (figs. 3 and 4 and table 1) but conserving practically intact the TNFSF-dependent extrinsic apoptotic pathway that already existed prior to that split. On the contrary, cell-death mechanisms regulated by TNFSFs have become significantly simplified in jawless fishes (figs. 3 and 4).

This study opens several interesting research paths: First, it remains to be determined the precise relationships among invertebrate and vertebrate TNFSF genes, given that the hitherto available analyses are quite superficial. In fact, any comparison that could have been attempted was hampered by not knowing the full complexity of the TNF superfamily in vertebrates, described here for the first time. For instance, it is possible that some invertebrate TNFSFs are more similar to the fish-specific genes characterized here than to the mammalian TNFSFs that are generally chosen to compare. Second, it is now possible to analyze in parallel the evolution of the TNF superfamily and the evolution of the TNF receptor superfamily, in order to determine whether these two gene families are indeed coevolving in vertebrate species, as some authors have suggested. Third, the large number of new TNFSF-fish genes described here and the fact that some fish species have a TNF superfamily that is more complex than the one found in humans and other mammals deserves further analyses. Finally, this study allows for comparative studies of the biological roles of TNFSF genes and gene products in distantly related vertebrate lineages. Given the fundamental roles of the TNFSF/TNFRSF system in immunity, these comparisons may provide useful clues to understand how the transition from innate to adaptive immune systems occurred prior to vertebrate emergence or how exactly the different adaptive systems found in agnathans and gnathostomes came into place.

## Materials and Methods

### Sequence Retrieval and Alignment

The TNFSF genes present in the genomes of nine fishes, the agnathans *E. burgeri* (inshore hagfish), *Let. camtschaticum* (arctic lamprey), and *P. marinus* (sea lamprey), the chondrichthyans *Rhindocon typus* (whale shark) and *Callorhinchus milii* (elephant shark), the actinopterygians *D. rerio* (zebrafish), *T. rubripes* (torafugu), *Lep. oculatus* (spotted gar), and the sarcopterygian *L. chalumnae* (coelacanth) were characterized. To do so, the sequences of those species in the nr, wgs, est, tsa, htgs, and gss databases at the National Center for Biotechnology Information (NCBI; http://ncbi.nlm.nih.gov; last accessed July 10, 2020) were explored, as follows. First, TBlastN searches with default parameters, using as queries the protein sequences corresponding to all human TNFSF genes, were performed. This allowed detecting a large number of putative TNFSF sequences of the nine fish species. After eliminating duplicates, partial sequences and some distantly related TNF-like sequences such as those of the C1q/TNF-related proteins (CTRP family; Wong et al. 2004), all the remnant sequences were again used as queries in TBlastN searches against the same databases until results became saturated, that is, no additional TNFSF sequences were detectable. All the sequences thus obtained plus the human ones were aligned using ClustalX 2.1 (Larkin et al. 2007) with default parameters and the alignment manually refined using GeneDoc 2.7 (Nicholas and Nicholas 1997).

### Phylogenetic Analyses

They closely followed the scheme described in a previous study (Marín 2018). IQTREE version 1.6.1 (Nguyen et al. 2015) was used to obtain ML phylogenetic trees based on the multiple-sequence alignments. The best model for amino acidic substitutions was obtained using ModelFinder (Kalyaanamoorthy et al. 2017). Following the recommendations of recent works (Nguyen et al. 2015; Zhou et al. 2018), ten replicates were obtained with three alternative perturbation strengths (0.2, 0.5, or 0.8) and 500 iterations of the nearest-neighbor interchange algorithm. The best ML tree obtained after all those analyses was selected. Ultrafast bootstrap analyses (1,000 replicates; Minh et al. 2013; Hoang et al. 2018) were performed to characterize the reliability of the topologies determined in the ML analyses. Branches with bootstrap values of at least 95% are considered to have a significant statistical support (Minh et al. 2013).

### Synteny Determination

The assembled genomes available at NCBI were analyzed to characterize whether the regions in which TNFSF genes were located in different species were syntenic. Often, multiple adjacent orthologous genes were detected in the same relative positions in several genomes, a robust proof for synteny. However, in other cases only one or a few genes adjacent to the TNF-encoding ones were detected as orthologous in different species. Given that the probability of finding in a couple of species that at least one of the genes that flank the TNFSF gene that is being analyzed is exactly in the same place by chance, and not by common ancestry, is very low (e.g., *p* = 1/5,000 in a genome with 20,000 genes), the presence of one or more genes immediately adjacent to a TNF-encoding gene that were orthologous in two different species was considered proof that the genes were located in syntenic regions. In the cases were no such synteny was detected, it was explored whether the TNFSF genes and their neighbors were positioned in the different species in regions of the same chromosomes, suggesting that intrachromosomal rearrangements (e.g., inversions) may have altered their location.

The quality of the current genomic assemblies determined several distinct strategies to establish orthologies and synteny among species. It was detected that the currently available assemblies for *Homo*, *Danio*, *Fugu*, *Lepisosteus*, *Latimeria*, and *Callorhinchus* are of high quality, meaning that the regions containing TNFSF genes that remain as isolated contigs and cannot be explored are exceptional. On the other hand, the quality of the assembly of the *Rhincodon* genome is not as good, meaning that some negative results (i.e., synteny not determined) were due to lack of data. To compensate for this fact, the genome of a closely related species, the great white shark *Carcharodon carcharias*, was also explored, in order to detect in it the regions missing in *Rhincodon*. Finally, the genomes of the three agnathans are still more fractioned and gene annotation is extremely poor. For this reason, the contigs of these species were examined in very precise detail using the NCBI tool ORFFinder (https://www.ncbi.nlm.nih.gov/orffinder/; last accessed July 10, 2020). About 200 kb (up to 1 Mb in regions with low density of genes) at each side of a given TNFSF sequence were analyzed. All potential ORFs of at least 25 amino acids detected by ORFFinder were checked using BlastP searches against the nr protein database at NCBI. In this way, the agnathan genes adjacent to the TNFSF members were characterized and, in several cases, synteny with the genomes of other species, established (see Results). Additional synteny information for species other than the ones already mentioned was obtained from the Genomicus website (Nguyen et al. 2018; http://www.genomicus.biologie.ens.fr/; last accessed July 10, 2020; database version 96.01).

### Impact of WGDs

To interpret whether some TNF duplications were linked to the WGDs that occurred in early vertebrate evolution, data from Sacerdot et al. (2018) were used (they are available at the Genomicus website; http://www.genomicus.biologie.ens.fr/genomicus-69.10; last accessed July 10, 2020). That work compared the genomes of current species to deduce the chromosomal complement of the vertebrate ancestor before those WGDs. Inversely, the genomes of modern species can be analyzed to establish from which of those ancestral chromosomes derived particular regions and, thus, whether multiple members of a gene family are located in paralogous regions derived from WGDs (paralogons) and therefore all may derive from a single progenitor gene.

## Supplementary Material

Supplementary data are available at *Genome Biology and Evolution* online.

## Supplementary Material

evaa140_Supplementary_Data

## References

[evaa140-B1] Aggarwal BB. 2003 Signalling pathways of the TNF superfamily: a double-edged sword. Nat Rev Immunol. 3(9):745–756. 12949498 10.1038/nri1184

[evaa140-B2] Aggarwal BB Gupta SC Kim JH. 2012 Historical perspectives on tumor necrosis factor and its superfamily: 25 years later, a golden journey. Blood 119(3):651–665. 22053109 10.1182/blood-2011-04-325225 PMC3265196

[evaa140-B3] Andersen DS, et al 2015 The *Drosophila* TNF receptor Grindelwald couples loss of cell polarity and neoplastic growth. Nature 522(7557):482–486. 25874673 10.1038/nature14298

[evaa140-B4] Betancur-R R, et al 2017 Phylogenetic classification of bony fishes. BMC Evol Biol. 17(1):162. 28683774 10.1186/s12862-017-0958-3 PMC5501477

[evaa140-B5] Beutler B Van Huffel C. 1994 An evolutionary and functional approach to the TNF receptor/ligand family. Ann N Y Acad Sci. 730:118–133. 8080166 10.1111/j.1749-6632.1994.tb44244.x

[evaa140-B6] Biswas G Kinoshita S Kono T Hikima J Sakai M. 2015 Evolutionary evidence of tumor necrosis factor super family members in the Japanese pufferfish (*Takifugu rubripes*): comprehensive genomic identification and expression analysis. Mar Genomics. 22:25–36. 25792259 10.1016/j.margen.2015.03.003

[evaa140-B7] Bodmer JL Schneider P Tschopp J. 2002 The molecular architecture of the TNF superfamily. Trends Biochem Sci. 27(1):19–26. 11796220 10.1016/s0968-0004(01)01995-8

[evaa140-B8] Boehm T, et al 2018 Evolution of alternative adaptive immune systems in vertebrates. Annu Rev Immunol. 36(1):19–42. 29144837 10.1146/annurev-immunol-042617-053028

[evaa140-B9] Borst J Hendriks J Xiao Y. 2005 CD27 and CD70 in T cell and B cell activation. Curr Opin Immunol. 17(3):275–281. 15886117 10.1016/j.coi.2005.04.004

[evaa140-B10] Braasch I, et al 2016 The spotted gar genome illuminates vertebrate evolution and facilitates human-teleost comparisons. Nat Genet. 48(4):427–437. 26950095 10.1038/ng.3526 PMC4817229

[evaa140-B11] Browning JL, et al 1993 Lymphotoxin beta, a novel member of the TNF family that forms a heteromeric complex with lymphotoxin on the cell surface. Cell 72(6):847–856. 7916655 10.1016/0092-8674(93)90574-a

[evaa140-B12] Collette Y Gilles A Pontarotti P Olive D. 2003 A co-evolution perspective of the TNFSF and TNFRSF families in the immune system. Trends Immunol. 24(7):387–394. 12860530 10.1016/s1471-4906(03)00166-2

[evaa140-B13] Dalloul RA, et al 2010 Multi-platform next-generation sequencing of the domestic turkey (*Meleagris gallopavo*): genome assembly and analysis. PLoS Biol. 8(9):pii: e1000475. 20838655 10.1371/journal.pbio.1000475 PMC2935454

[evaa140-B14] Das S, et al 2016 Characterization of lamprey BAFF-like gene: evolutionary implications. J Immunol. 197(7):2695–2703. 27543613 10.4049/jimmunol.1600799 PMC5026938

[evaa140-B15] Delsuc F Brinkmann H Chourrout D Philippe H. 2006 Tunicates and not cephalochordates are the closest living relatives of vertebrates. Nature 439(7079):965–968. 16495997 10.1038/nature04336

[evaa140-B16] Delsuc F Tsagkogeorga G Lartillot N Philippe H. 2008 Additional molecular support for the new chordate phylogeny. Genesis 46(11):592–604. 19003928 10.1002/dvg.20450

[evaa140-B17] Delsuc F, et al 2018 A phylogenomic framework and timescale for comparative studies of tunicates. BMC Biol. 16(1):39. 29653534 10.1186/s12915-018-0499-2 PMC5899321

[evaa140-B18] Dostert C Grusdat M Letellier E Brenner D. 2019 The TNF family of ligands and receptors: communication modules in the immune system and beyond. Physiol Rev. 99(1):115–160. 30354964 10.1152/physrev.00045.2017

[evaa140-B19] Elgueta R, et al 2009 Molecular mechanism and function of CD40/CD40L engagement in the immune system. Immunol Rev. 229(1):152–172. 19426221 10.1111/j.1600-065X.2009.00782.x PMC3826168

[evaa140-B20] Feuda R, et al 2017 Improved modeling of compositional heterogeneity supports sponges as sister to all other animals. Curr Biol. 27(24):3864–3870. 29199080 10.1016/j.cub.2017.11.008

[evaa140-B21] Flajnik MF. 2018 A cold-blooded view of adaptive immunity. Nat Rev Immunol. 18(7):438–453. 29556016 10.1038/s41577-018-0003-9 PMC6084782

[evaa140-B22] Gao D, et al 2015 Repertoire and evolution of TNF superfamily *in Crassostrea gigas*: implications for expansion and diversification of this superfamily in Mollusca. Dev Comp Immunol. 51(2):251–260. 25910814 10.1016/j.dci.2015.04.006

[evaa140-B23] Gilmore TD Wolenski FS. 2012 NF-κB: where did it come from and why? Immunol Rev. 246(1):14–35. 22435545 10.1111/j.1600-065X.2012.01096.x

[evaa140-B24] Glenney GW Wiens GD. 2007 Early diversification of the TNF superfamily in teleosts: genomic characterization and expression analysis. J Immunol. 178(12):7955–7973. 17548633 10.4049/jimmunol.178.12.7955

[evaa140-B25] Hehlgans T Pfeffer K. 2005 The intriguing biology of the tumour necrosis factor/tumour necrosis factor receptor superfamily: players, rules and the games. Immunology 115(1):1–20. 15819693 10.1111/j.1365-2567.2005.02143.x PMC1782125

[evaa140-B26] Hoang DT, et al 2018 MPBoot: fast phylogenetic maximum parsimony tree inference and bootstrap approximation. BMC Evol Biol. 18(1):11. 29390973 10.1186/s12862-018-1131-3 PMC5796505

[evaa140-B27] Hong S Li R Xu Q Secombes CJ Wang T. 2013 Two types of TNF-α exist in teleost fish: phylogeny, expression, and bioactivity analysis of type-II TNF-α3 in rainbow trout *Oncorhynchus mykiss*. J Immunol. 191(12):5959–5972. 24244011 10.4049/jimmunol.1301584

[evaa140-B28] Huang S, et al 2008 Genomic analysis of the immune gene repertoire of amphioxus reveals extraordinary innate complexity and diversity. Genome Res. 18(7):1112–1126. 18562681 10.1101/gr.069674.107 PMC2493400

[evaa140-B29] Hughes LC, et al 2018 Comprehensive phylogeny of ray-finned fishes (*Actinopterygii*) based on transcriptomic and genomic data. Proc Natl Acad Sci U S A. 115(24):6249–6254. 29760103 10.1073/pnas.1719358115 PMC6004478

[evaa140-B30] Igaki T Miura M. 2014 The *Drosophila* TNF ortholog *Eiger*: emerging physiological roles and evolution of the TNF system. Semin Immunol. 26(3):267–274. 24981286 10.1016/j.smim.2014.05.003

[evaa140-B31] Irisarri I, et al 2017 Phylotranscriptomic consolidation of the jawed vertebrate timetree. Nat Ecol Evol. 1(9):1370–1378. 28890940 10.1038/s41559-017-0240-5 PMC5584656

[evaa140-B32] Kaiser P, et al 2005 A genomic analysis of chicken cytokines and chemokines. J Interferon Cytokine Res. 25(8):467–484. 16108730 10.1089/jir.2005.25.467

[evaa140-B33] Kalyaanamoorthy S Minh BQ Wong TKF von Haeseler A Jermiin LS. 2017 ModelFinder: fast model selection for accurate phylogenetic estimates. Nat Methods. 14(6):587–589. 28481363 10.1038/nmeth.4285 PMC5453245

[evaa140-B34] Kasahara M. 1998 What do the paralogous regions in the genome tell us about the origin of the adaptive immune system? Immunol Rev. 166(1):159–175. 9914911 10.1111/j.1600-065x.1998.tb01261.x

[evaa140-B35] Kaufman J. 2018 Unfinished business: evolution of the MHC and the adaptive immune system of jawed vertebrates. Annu Rev Immunol. 36(1):383–409. 29677478 10.1146/annurev-immunol-051116-052450

[evaa140-B36] Kauppila S, et al 2003 *Eiger* and its receptor, Wengen, comprise a TNF-like system in *Drosophila*. Oncogene 22(31):4860–4867. 12894227 10.1038/sj.onc.1206715

[evaa140-B37] Kinoshita S Biswas G Kono T Hikima J Sakai M. 2014 Presence of two tumor necrosis factor (TNF)-α homologs on different chromosomes of zebrafish (*Danio rerio*) and medaka (*Oryzias latipes*). Mar Genomics. 13:1–9. 24269726 10.1016/j.margen.2013.10.004

[evaa140-B38] Kuraku S Meyer A Kuratani S. 2009 Timing of genome duplications relative to the origin of the vertebrates: did cyclostomes diverge before or after? Mol Biol Evol. 26(1):47–59. 18842688 10.1093/molbev/msn222

[evaa140-B39] Larkin MA, et al 2007 Clustal W and Clustal X version 2.0. Bioinformatics 23(21):2947–2948. 17846036 10.1093/bioinformatics/btm404

[evaa140-B40] Legrand AJ Konstantinou M Goode EF Meier P. 2019 The diversification of cell death and immunity: memento mori. Mol Cell 76(2):232–242. 31586546 10.1016/j.molcel.2019.09.006

[evaa140-B41] Li R Dooley H Wang T Secombes CJ Bird S. 2012 Characterisation and expression analysis of B-cell activating factor (BAFF) in spiny dogfish (*Squalus acanthias*):cartilaginous fish BAFF has a unique extra exon that may impact receptor binding. Dev Comp Immunol. 36(4):707–717. 22155638 10.1016/j.dci.2011.11.010

[evaa140-B42] Li R, et al 2015 Characterisation of the TNF superfamily members *CD40L* and *BAFF* in the small-spotted catshark (*Scyliorhinus canicula*). Fish Shellfish Immunol. 47(1):381–389. 26386192 10.1016/j.fsi.2015.09.033

[evaa140-B43] Lobito AA Gabriel TL Medema JP Kimberley FC. 2011 Disease causing mutations in the TNF and TNFR superfamilies: focus on molecular mechanisms driving disease. Trends Mol Med. 17(9):494–505. 21724465 10.1016/j.molmed.2011.05.006

[evaa140-B44] Locksley RM Killeen N Lenardo MJ. 2001 The TNF and TNF receptor superfamilies: integrating mammalian biology. Cell 104(4):487–501. 11239407 10.1016/s0092-8674(01)00237-9

[evaa140-B45] Maeda T Suetake H Odaka T Miyadai T. 2018 Original ligand for LTβR Is LIGHT: insight into evolution of the LT/LTβR system. J Immunol. 201(1):202–214. 29769272 10.4049/jimmunol.1700900

[evaa140-B46] Marín I. 2018 Origin and evolution of fungal HECT ubiquitin ligases. Sci Rep. 8(1):6419. 29686411 10.1038/s41598-018-24914-x PMC5913265

[evaa140-B47] Minh BQ Nguyen MA von Haeseler A. 2013 Ultrafast approximation for phylogenetic bootstrap. Mol Biol Evol. 30(5):1188–1195. 23418397 10.1093/molbev/mst024 PMC3670741

[evaa140-B48] Moreno E Yan M Basler K. 2002 Evolution of TNF signaling mechanisms: JNK-dependent apoptosis triggered by *Eiger*, the *Drosophila* homolog of the TNF superfamily. Curr Biol. 12(14):1263–1268. 12176339 10.1016/s0960-9822(02)00954-5

[evaa140-B49] Near TJ, et al 2012 Resolution of ray-finned fish phylogeny and timing of diversification. Proc Natl Acad Sci U S A. 109(34):13698–13703. 22869754 10.1073/pnas.1206625109 PMC3427055

[evaa140-B50] Nicholas KB Nicholas HB Jr. 1997. GeneDoc; a tool for editing and annotating multiple sequence alignments. Distributed by the authors.

[evaa140-B51] Nguyen LT Schmidt HA von Haeseler A Minh BQ. 2015 IQ-TREE: a fast and effective stochastic algorithm for estimating maximum-likelihood phylogenies. Mol Biol Evol. 32(1):268–274. 25371430 10.1093/molbev/msu300 PMC4271533

[evaa140-B52] Nguyen NTT Vincens P Roest Crollius H Louis A. 2018 Genomicus 2018: karyotype evolutionary trees and on-the-fly synteny computing. Nucleic Acids Res. 46(D1):D816–D822. 29087490 10.1093/nar/gkx1003 PMC5753199

[evaa140-B53] Nocentini G Riccardi C. 2009 GITR: a modulator of immune response and inflammation. Adv Exp Med Biol. 647:156–173. 19760073 10.1007/978-0-387-89520-8_11

[evaa140-B54] Pantalacci S, et al 2008 Conserved features and evolutionary shifts of the EDA signaling pathway involved in vertebrate skin appendage development. Mol Biol Evol. 25(5):912–928. 18304980 10.1093/molbev/msn038

[evaa140-B55] Parrinello N Cammaratta M Parrinello D. 2018 The inflammatory response of Urochordata: the basic process of the Ascidians’ innate immunity In: Cooper EI, editor. Advances in comparative immunology. Cham, Switzerland: Springer International Publishing AG. p. 521–590.

[evaa140-B56] Pozzolini M, et al 2016 Molecular characterization and expression analysis of the first Porifera tumor necrosis factor superfamily member and of its putative receptor in the marine sponge *Chondrosia reniformis*. Dev Comp Immunol. 57:88–98. 26705701 10.1016/j.dci.2015.12.011

[evaa140-B57] Premzl M. 2016 Comparative genomic analysis of eutherian tumor necrosis factor ligand genes. Immunogenetics 68(2):125–132. 26646413 10.1007/s00251-015-0887-5

[evaa140-B58] Qin N Tang T Liu X Xie S Liu F. 2019 Involvement of a TNF homologue in balancing the host immune system of *Macrobrachium nipponense*. Int J Biol Macromol. 134:73–79. 31075328 10.1016/j.ijbiomac.2019.05.045

[evaa140-B59] Quistad SD Traylor-Knowles N. 2016 Precambrian origins of the TNFR superfamily. Cell Death Discov. 2:16058. 27551546 10.1038/cddiscovery.2016.58 PMC4979521

[evaa140-B60] Quistad SD, et al 2014 Evolution of TNF-induced apoptosis reveals 550 My of functional conservation. Proc Natl Acad Sci U S A. 111(26):9567–9572. 24927546 10.1073/pnas.1405912111 PMC4084427

[evaa140-B61] Ravi V Venkatesh B. 2018 The divergent genomes of teleosts. Annu Rev Anim Biosci. 6(1):47–68. 29447475 10.1146/annurev-animal-030117-014821

[evaa140-B62] Redmond AK Pettinello R Dooley H. 2017 Outgroup, alignment and modelling improvements indicate that two *TNFSF13*-like genes existed in the vertebrate ancestor. Immunogenetics 69(3):187–192. 28070614 10.1007/s00251-016-0967-1 PMC5316386

[evaa140-B63] Ren W Pang S You F Zhou L Zhang S. 2011 The first *BAFF* gene cloned from the cartilaginous fish. Fish Shellfish Immunol. 31(6):1088–1096. 21959037 10.1016/j.fsi.2011.09.013

[evaa140-B64] Richter DJ Fozouni P Eisen MB King N. 2018 Gene family innovation, conservation and loss on the animal stem lineage. Elife 7:pii: e34226. 29848444 10.7554/eLife.34226 PMC6040629

[evaa140-B65] Riesgo A Farrar N Windsor PJ Giribet G Leys SP. 2014 The analysis of eight transcriptomes from all poriferan classes reveals surprising genetic complexity in sponges. Mol Biol Evol. 31(5):1102–1120. 24497032 10.1093/molbev/msu057

[evaa140-B66] Robertson AJ, et al 2006 The genomic underpinnings of apoptosis in *Strongylocentrotus purpuratus*. Dev Biol. 300(1):321–334. 17010332 10.1016/j.ydbio.2006.08.053

[evaa140-B67] Rohde F, et al 2018 Characterization of chicken tumor necrosis factor-α, a long missed cytokine in birds. Front Immunol. 9:605. 29719531 10.3389/fimmu.2018.00605 PMC5913325

[evaa140-B68] Roschke V, et al 2002 BLyS and APRIL form biologically active heterotrimers that are expressed in patients with systemic immune-based rheumatic diseases. J Immunol. 169(8):4314–4321. 12370363 10.4049/jimmunol.169.8.4314

[evaa140-B69] Ruvkun G Hobert O. 1998 The taxonomy of developmental control in *Caenorhabditis elegans*. Science 282(5396):2033–2041. 9851920 10.1126/science.282.5396.2033

[evaa140-B70] Sacerdot C Louis A Bon C Berthelot C Roest Crollius H. 2018 Chromosome evolution at the origin of the ancestral vertebrate genome. Genome Biol. 19(1):166. 30333059 10.1186/s13059-018-1559-1 PMC6193309

[evaa140-B71] Savan R Kono T Igawa D Sakai M. 2005 A novel tumor necrosis factor (TNF) gene present in tandem with the TNF-alpha gene on the same chromosome in teleosts. Immunogenetics 57(1–2):140–150. 15759114 10.1007/s00251-005-0768-4

[evaa140-B72] Smith JJ, et al 2013 Sequencing of the sea lamprey (*Petromyzon marinus*) genome provides insights into vertebrate evolution. Nat Genet. 45(4):415–421. 23435085 10.1038/ng.2568 PMC3709584

[evaa140-B73] Smith JJ, et al 2018 The sea lamprey germline genome provides insights into programmed genome rearrangement and vertebrate evolution. Nat Genet. 50(2):270–277. 29358652 10.1038/s41588-017-0036-1 PMC5805609

[evaa140-B74] Suzuki T Shin-I T Kohara Y Kasahara M. 2004 Transcriptome analysis of hagfish leukocytes: a framework for understanding the immune system of jawless fishes. Dev Comp Immunol. 28(10):993–1003. 15236930 10.1016/j.dci.2004.04.005

[evaa140-B75] Tacchi L Larragoite ET Muñoz P Amemiya CT Salinas I. 2015 African lungfish reveal the evolutionary origins of organized mucosal lymphoid tissue in vertebrates. Curr Biol. 25(18):2417–2424. 26344090 10.1016/j.cub.2015.07.066 PMC4869758

[evaa140-B76] van der Weyden CA Pileri SA Feldman AL Whisstock J Prince HM. 2017 Understanding CD30 biology and therapeutic targeting: a historical perspective providing insight into future directions. Blood Cancer J. 7(9):e603. 28885612 10.1038/bcj.2017.85 PMC5709754

[evaa140-B77] Vanamee ÉS Faustman DL. 2018 Structural principles of tumor necrosis factor superfamily signaling. Sci Signal. 11(511):pii: eaao4910. 29295955 10.1126/scisignal.aao4910

[evaa140-B78] Venkatesh B, et al 2014 Elephant shark genome provides unique insights into gnathostome evolution. Nature 505(7482):174–179. 24402279 10.1038/nature12826 PMC3964593

[evaa140-B79] Wallach D. 2018 The Tumor Necrosis Factor family: family conventions and private idiosyncrasies. Cold Spring Harb Perspect Biol. 10(10):pii: a028431. 28847899 10.1101/cshperspect.a028431 PMC6169814

[evaa140-B80] Wang PH, et al 2012 Molecular cloning, characterization and expression analysis of the tumor necrosis factor (TNF) superfamily gene, TNF receptor superfamily gene and lipopolysaccharide-induced TNF-α factor (LITAF) gene from *Litopenaeus vannamei*. Dev Comp Immunol. 36(1):39–50. 21736897 10.1016/j.dci.2011.06.002

[evaa140-B81] Wiens GD Glenney GW. 2011 Origin and evolution of TNF and TNF receptor superfamilies. Dev Comp Immunol. 35(12):1324–1335. 21527275 10.1016/j.dci.2011.03.031

[evaa140-B82] Wong GW Wang J Hug C Tsao TS Lodish HF. 2004 A family of Acrp30/adiponectin structural and functional paralogs. Proc Natl Acad Sci U S A. 101(28):10302–10307. 15231994 10.1073/pnas.0403760101 PMC478567

[evaa140-B83] Yamaguchi K, et al 1998 Characterization of structural domains of human osteoclastogenesis inhibitory factor. J Biol Chem. 273(9):5117–5123. 9478964 10.1074/jbc.273.9.5117

[evaa140-B84] Yi F Frazzette N Cruz AC Klebanoff CA Siegel RM. 2018 Beyond cell death: new functions for TNF family cytokines in autoimmunity and tumor immunotherapy. Trends Mol Med. 24(7):642–653. 29880309 10.1016/j.molmed.2018.05.004 PMC7466867

[evaa140-B85] Zhang G. 2004 Tumor necrosis factor family ligand-receptor binding. Curr Opin Struct Biol. 14(2):154–160. 15093829 10.1016/j.sbi.2004.03.003

[evaa140-B86] Zhang JY, et al 2010 The genomic underpinnings of apoptosis in the silkworm, *Bombyx mori*. BMC Genomics. 11:611. 21040523 10.1186/1471-2164-11-611 PMC3091752

[evaa140-B87] Zhang Q Lenardo MJ Baltimore D. 2017 30 years of NF-κB: a blossoming of relevance to human pathobiology. Cell 168(1–2):37–57. 28086098 10.1016/j.cell.2016.12.012 PMC5268070

[evaa140-B88] Zhang X Luan W Jin S Xiang J. 2008 A novel tumor necrosis factor ligand superfamily member (CsTL) from *Ciona savignyi*: molecular identification and expression analysis. Dev Comp Immunol. 32(11):1362–1373. 18579203 10.1016/j.dci.2008.05.009

[evaa140-B89] Zhao Y, et al 2019 Cambrian sessile, suspension feeding stem-group ctenophores and evolution of the comb jelly body plan. Curr Biol. 29(7):1112–1125. 30905603 10.1016/j.cub.2019.02.036

[evaa140-B90] Zhou X Shen XX Hittinger CT Rokas A. 2018 Evaluating fast maximum likelihood-based phylogenetic programs using empirical phylogenomic data sets. Mol Biol Evol. 35(2):486–503. 29177474 10.1093/molbev/msx302 PMC5850867

