# Supplementary material for: Tumor Necrosis Factor Superfamily: Ancestral Functions and Remodeling in Early Vertebrate Evolution
Source: Genome Biol Evol. 2020 Jul 6;12(11):2074–92. doi: 10.1093/gbe/evaa140 (PMC7674686; doi:10.1093/gbe/evaa140)
Supplement: evaa140_Supplementary_Data [file evaa140_supplementary_data.zip › Supplementary file 2.pdf]

Along this file: *genes in blue are adjacent in humans to the corresponding TNFSF genes. Genes in red are located in humans in the same chromosome.*
In parentheses, the chromosome/chromosomal region in which the corresponding human ortholog of a fish gene is found
N. a.: information not available, due to lack of data
[WGD]: derived from the teleost-specific whole genome duplication
Dots: synteny deduced from the data, see Figure 1 legend in the main text for details

---

TNFSF1/2/3 (Homo: 6p21.33) - TNFSF-NEW (Danio, Takifugu) - Tandem duplicates in sharks

Homo: **ATP6V1G2 - NFKBIL1 – TNFSF1 – TNFSF2 – TNFSF3 – LST1 – NCR3** ●

Latimeria: **ZBTB12 (6p21.33)- C2 (6p21.33) - XM_006007194.2/TNFSF1 – XM_006007185.2/TNFSF2 – XM_014495272.1/TNFSF3** – RGS3 (9) - PTGES (9) ●
N. a. - **XM_006014356./TNFSF2-like** – N. a.

Lepisosteus: N. a. - **XM_015339713.1/TNFSF1/2 – NEU1 (6p21.33)** ○

Danio: TSPAN13 (7) - ? - **BAG6 (6p21.33)** - **XM_009291801.3/TNFSF-NEW - NM_001024447.1/TNFSF1/2 - GABBR1 (6p22.1) -** SH3BP5L (1) ○
ZBTB26 (9) - SACM1L (3) - **NM_212859.2/TNFSF1/2-like - GABBR1 (6p22.1)** - SH3BP5L (1) **[WGD]** ○

Takifugu: GLRX3 (10) - **APOM (6p21.33)** - **NM_001037986.1/TNFSF-NEW** - **NM_001037985.1/TNFSF1/2** - **GABBR1 (6p22.1) -** SH3BP5L (1) ○

Rhincodon:
N. a. **- 6 x tandem TNFSF1/2** – N. a.
N. a. **- XM_020516657.1/TNFSF1/2-like** – N. a.
N. a. **- LVEK02020348.1/TNFSF1/2-like** – N. a.

Carcharodon QUOW01004891.1: N. a. **- 6 x tandem TNFSF1/2 – TRIM39 (6p22.1) - BTN2A2 (6p22.2)** ○

Callorhinchus: all absent

Carcharodon data validates synteny for *Rhincodon* genes
ATP6V1G1/G2 very close to, respectively, TNFSF15 and TNFSF1/2/3

TNFSF15/8 (Homo 9q32) - TNFSF-Fish2 - TNFSF-Fish5

Homo: **DELEC1 – TNC – TNFSF8 – TNFSF15 – TEX48 - TEX53 - TMEM268 - ATP6V1G1** ●

Latimeria:
N. a - **XM_014498535.1/TNFSF15 - XM_006012363.2/TNFSF-Fish2 –** N. a. ○
N. a - **XM_006010739.2/TNFSF8 –** N. a.
N. a. **– BAHO01042088.1/TNFSF-Fish5 –** N. a.

Lepisosteus: P2RY2 (11) - ? - **MEGF9 (9q33.2)** - **XM_015367164.1/TNFSF15 - XR_001480777.1/TNFSF-Fish5 - EDF1 (9q34.3) – CIZ1 (9q34.11)** ○

Danio: **GOLGA1 (9q33.3) - MEGF9 (9q33.2)** - **XM_005169085.4/TNFSF15 –** GFRA2 (8) - DOK2 (8) ○

Callorhinchus:
RGS3 (9q32) - MEGF9 (9q33.2) - *XM_007909646.1/TNFSF-like* - **XM_007909635.1/TNFSF15 – NM_001292244.1/TNFSF-Fish2 -**
- XM_007909644.1/TNFSF-Fish5 - TMEM268 - ATP6V1G1 ●

N. a - **AAVX02013886.1/TNFSF15-2 –** N. a.

Rhincodon:
N. a. - **XM_020514766.1/TNFSF-Fish2 – XM_020514765.1/TNFSF-Fish5 –** N. a.
N. a. **– XM_020531692.1/TNFSF15 –** N. a.
N. a. – **LVEK02012960.1/TNFSF15-2** – N. a.

***Carcharodon** QUOW01009139.1:* **MEGF9 (9q33.2)** - *TNFSF-like* **–TNFSF15 – TNFSF-Fish2 – TNFSF-Fish5 - TMEM268 - ATP6V1G1** ●

Takifugu: all absent

Callorhinchus XM_007909646.1/TNFSF-like was not included in ths study. Due to very low similarity, it was discarded as a partial sequence. It shows some similarity to particular bird TNFSFs.

Carcharodon data validates synteny for *Rhincodon* genes

ATP6V1G1 and ATP6V1G2 very close to, respectively, TNFSF15 and TNFSF1/2/3

EDA (Homo: Xq13.1) - BALM

Homo: PJA1 - FAM155B – EDA - AWAT2 - OTUD6A – IGBP1 ●

Latimeria: EFNB1 (Xq13.1) - FAM155B – BALM - EDA - LRRC32 (11) - WNT11 (11) – IGBP1 ●

Lepisosteus: EFNB1 (Xq13.1) - FAM155B – EDA – BALM - LRRC32 (11) - WNT11 (11) – IGBP1 ●

Danio: EFNB1 (Xq13.1) - FAM155B – EDA – WNT11 (11) – IGBP1 ●

Takifugu: NLGN3 (Xq13.1) - GRIA3 (Xq25) - EDA – BALM – LRRC32 (11) – TDRD1 (10) ○ (EDA) ● (BALM)

Callorhinchus:
{ INPPL1 (11) – ARR3 (Xq13.1) - EDA - COL4A6 (Xq22.3) - COL4A5 (Xq22.3) ○
N. a. – BALM – N. a.

Rhincodon:
{ N. a. – EDA – N. a.
ATG4A (Xq22.3) - BALM – N. a. ○

Carcharodon QUOW01007644.1 { ARR3 (Xq13.1) - FAM155B – EDA - COL4A6 (Xq22.3) – KXD1 (19) ●
QUOW01001656.1 PSMD10 (Xq22.3) – ATG4A (Xq22.3) – BALM – N. a. ○

Carcharodon data validates synteny for *Rhincodon* genes

FAM155B and a gene similar to FAM155A are close to, respectively, EDA/BALM and TNFSF13B

Eptatretus FYBX02010013.1: ST8SIA3 (18) – ST6GALNAC1 (17q25.1) – **EDA-like** – CHP1 (15) – TTC8 (14)

Petromyzon PIZI01000055.1: ATP6AP2 (Xp11.4) - GUCY2F (Xq22.3) - UBXN7 (3q29) – COPS9 (2) – **EDA-like** – GRK1 (13q34) – METTL16 (17p13.3) – ATP1B2 (17p13.1)

Eptatretus FYBX02010170.1: ZBED9 (6) – HTR3A (11) – **TNFSF x 3** – MTSS1 (8) – LOC100287896 (11) – AGA (4)

Petromyzon KX146631.1/PIZI01000373.1: N. a. – **TNFSF** – IGBP1 – N. a.

TNFSF12 - TNFSF13 (Homo: 17p13.1)

Homo: SLC35G6 - POLR2A – TNFSF12 – TNFSF13 – SENP3 - EIF4A1 ●

Latimeria: ZP2 (16) - LDLRAP1 (1) - **TNFSF12 – TNFSF13 – WRAP53 (17p13.1) – MINK1 (17p13.2)** ○

Lepisosteus:
- **KCNAB3 (17p13.1)** - TIPARP (3) - **TNFSF13 – DGL4 (17p13.1)** – EPHB4 (7) ○
- ZP2 (16) – ? - FAM43B (1) - **TNFSF12 – CHD3 (17p13.1) - CYB5D1 (17p13.1)** ○

Danio:
- **KCNAB3 (17p13.1) – KIF1C (17p13.2) – TNFSF13 - ?** - EPHB4 (7) **– DGL4 (17p13.1)** ○
- ZP2 (16) - ? - ? - **SAT2 (17p13.1) – SAT2 (17p13.1) – TNFSF12 – CHD3 (17p13.1) - CYB5D1 (17p13.1)** ○

Takifugu: MAN2B2 (4) - ? - ? - **SAT2 (17p13.1) – SAT2 (17p13.1) – TNFSF12 - CHD3 (17p13.1) – NAA38 (17p13.1)** ○

Callorhinchus:
- N. a. – **TNFSF12** – N. a.
- N. a. – **TNFSF13** – N. a.

Rhincodon: N. a. **– TNFSF12 – TNFSF13** – TMEM217 (6) – N. a.

Carcharodon:
QUOW01007215.1 **TRAPPC1 (17p13.1)** – LDLRAP1 (1) – **TNFSF12 – TNFSF13** – **WRAP53 (17p13.1) –** PBMUCL2 (6) ○

Carcharodon data validates synteny for *Rhincodon* genes

TNFSF13B (Homo: 13q33.3)

Homo: LIG4 – ABHD13 – **TNFSF13B** – MYO16 – IRS2 ●

Latimeria: LIG4 – ABHD13 – **TNFSF13B** – MYO16 – IRS2 ●

Lepisosteus: LIG4 – ABHD13 – **TNFSF13B** – MYO16 – IRS2 ●

Danio: FAM155A (9) - ABHD13 – **TNFSF13B** – ACVR1C (2) – CYTIP (2) ●

Takifugu: FAM155A (9) - ABHD13 – **TNFSF13B** – MYO16 – IRS2 ●

Callorhinchus: LIG4 – ABHD13 – **TNFSF13B** – MYO16 – IRS2 ●

Rhincodon:
- LIG4 – ABHD13 – **TNFSF13B/ XM_020515722.1** – N. a. ●
- WBP2 (17) – TIMM88 (11) – **TNFSF13B-2/XM_020528564.1** - SOCS1 (16)

FAM155B and a gene similar to FAM155A are close to, respectively, EDA/BALM and TNFSF13B
Rhincodon TNFSF13B-2/XM_020528564.1 is a potential transposed duplicate

---

Eptatretus FYBX02009995.1: EFNB2 (13q33.3) - ABHD13 – **TNFSF** – ARHGAP45 (19) – BARHL2 (1)

TNFSF9 – TNFSF7 – TNFSF14 (Homo: 19p13.3) + fish-specific tandem duplicates

Homo: DENND1C- TUBB4A – TNFSF9 – TNFSF7 – TNFSF14 – C3 – GPR108 ●

Latimeria:
{ N. a. - TNFSF14/XM_014487431.1 - TNFSF14/XM_014487432.1 – C3 – C3 – RDH8 (19p13.2) ●
N. a. – TNFSF9/XM_006013505.2 – N. a.

Lepisosteus: GJA8 (1) – PLPP3 (1) – TNFSF*/XM_015348906.1 – TNFSF14/AHAT01007520.1/pseudo - TNFSF14/AHAT01007520.1 – TNFSF14/XM_006631663.2 –
- TNFSF14 /XM_015348887.1 – TMED1 (19p13.2) – TNFSF14/XM_015349027.1 – DNM2 (19p13.2) - QTRT1 (19p13.2) ○

Danio: TRIM16 (17) - ADGRL1 (19p13.12) - ADGRL1 (19p13.12) - ADGRL1 (19p13.12) - TNFSF9/XM_009296078.3 - TNFSF14/XM_021475881.1 - TMED1 (19p13.2) - DNM2 (19p13.2) ○
ADGRL1 (19p13.12) – PLPP3 (1) - TNFSF*/NM_001128818.1 - TNFSF14-2/NM_001281995.1 - TMED1 (19p13.2) - DNM2 (19p13.2) [WGD] ○

Takifugu: NXNL1 (19p13.11) - ADGRL1 (19p13.12) - TNFSF9/XM_011612517.1 - TNFSF14/XM_003972088.1 – TMED1 (19p13.2) - DNM2 (19p13.2) ○

Rhincodon:
N. a. - TNFSF14/XM_020524650.1 – N. a.
N. a. - TNFSF9/LVEK02054556.1 – N. a.

Callorhinchus: N. a. - TNFSF9/AAVX02054119.1 – N. a.

Carcharodon data not available

Lepisosteus TNFSF*/XM_015348906.1, Danio TNFSF*/NM_001128818.1 are genes not assignable to any orthology group by sequence similarity

TMED1, TMED5 are close to, respectively TNFSF4/18/6 and TNFSF9/7/14

Eptatretus FYBX02009869.1: ALG13 (Xq23) - CCDC151 (19p13.2) - TNFSF - DNM2 (19p13.2) – SALL1 (16) – MED25 (19q13.3) – N. a.

Petromyzon PIZI01000010.1: DAO (12) - SSC5D (19q13.42) – TNFSF – SLC25A39 (17) - TLL2 (10) – C3 – C3 – C5 (9)

TNFSF4 – TNFSF18 - TNFSF6 (Homo: 1q24.3-1q25.1)

Homo: SLC9C2 - PRDX6 - TNFSF4 – TNFSF18 – AIMP1P2 – TNFSF6 – SUCO - PIGC

Latimeria: N. a. - XM_014489943.1/TNFSF6 – SUCO – TMED5 (1.p22.1)

Lepisosteus: GXK1 (1q25.2) - TADA1 (1q24.1) - XM_006635296.2/TNFSF6 – SUCO – TMED5 (1.p22.1)

Danio: JUN (1p32.1) - ANK2 (4) - NM_001042701.2/TNFSF6 – FAM20B (1q25.2) - RALGPS2 (1q25.2)

Takifugu: JUN (1p32.1) - ? - XM_003974097.2/TNFSF6 - FAM20B (1q25.2) - RALGPS2 (1q25.2)

Callorhinchus: PRDX6 - TADA1 (1q24.1) – XM_007895693.1/TNFSF4 – AAVX02013887.1-pseudo/TNFSF6 - NM_001292981.1/TNFSF6 - XM_007895611.1/TNFSF6 - XM_007895612.1/TNFSF6 - – SUCO – TMED5 (1.p22.1)

Rhincodon:
N. a. - XM_020530320.1/TNFSF6 - XM_020530325.1/TNFSF6 - XM_020530323.1/TNFSF6 – SUCO – TMED5 (1.p22.1)
N. a. – LVEK02054556.1/TNFSF4 – N. a.

TMED1, TMED5 are genes close to, resepctively TNFSF4/18/6 and TNFSF9/7/14

Eptatretus FYBX02009657.1: COLGALT2 (1q25.3) – RGL1 (1q25.3) - TNFSF – TIPRL (1q24.2) – NMNAT2 (1q25.3)

Petromyzon PIZI01000063.1: STXBP6 (14) – LHX8 (1p31.1) - TNFSF – NMNAT2 (1q25.3) – KCNT2 (1q31.3)

Petromyzon PIZI01000007.1: FUBP1 (1p31.1) – IER5 (1q25.3) - PTPA (9) - NXPE3 (3) - TNFSF x 2 – UAP1 (1q23.3) – CASZ1 (1p36.22)

TNFSF5 (Homo: Xq26.3)

Homo: HTATSF1 - VGLL1 – TNFSF5 - ARHGEF6 – RBMX ●

Latimeria: HTATSF1 - VGLL1 – XM_014485204.1/TNFSF5 - ARHGEF6 – RBMX ●

Lepisosteus: HTATSF1 - VGLL1 – OPN3 (1) - XM_006632995.2/TNFSF5 - ARHGEF6 – RBMX ●

Danio: ADGRG4 (Xq26.3) - VGLL1 – OPN3 (1) - NM_001144809.1/TNFSF5 - ARHGEF6 – RBMX ●

Takifugu: ADGRG4 (Xq26.3) - OPN3 (1) - XM_011610441.2/TNFSF5 - ARHGEF6 – RBMX ●

Callorhinchus: ADGRG4 (Xq26.3) - VGLL1 – XM_007892719.1/TNFSF5 - ARHGEF6 – RBMX ●

Rhincodon: absent

Eptatretus FYBX02009755.1: H2BC13 (6) - H1-2 (6) – H4C15 (1) – **TNFSF** – AP1S2 (Xp22.2) – MBNL3 (Xq26.2) – RBMX

TNFSF-Fish3 (Lepisosteus: LG7, region corresponding to human X)

Lepisosteus: ENOX2 (Xq26.1) – ABCC5 (3q27.1) – XM_015351627.1/TNFSF-Fish3 – MED12 (Xq13.1) – MXRA5 (Xp22.33) ●

Danio: GLRA2 (Xp22.2) – AIFM1 (Xq26.1) - XM_005165144.3/TNFSF-Fish3 – CASP10 (2) – NONO (Xq13.1) ○

Callorhinchus: ENOX2 (Xq26.1) – ABCC5 (3q27.1) – XM_007891514.1/TNFSF-Fish3 – FSIP2 (2) – SIAH2 (3) ●

Rhincodon: N. a. - ABCC5 (3q27.1) – XM_020520727.1/TNFSF-Fish3 – N. a. ●

Homo, Latimeria, Takifugu: absent

TNFSF11 (Homo: 13q14.11)

**Homo:**	**AKAP11 - FABP3P2 – TNFSF11 - FAM216B – EPSTI1 - DNAJC15** ●
**Latimeria:**	N. a. - **AKAP11 - XM_014497209.1/TNFSF11** – N. a. ●
**Lepisosteus:**	**DGKH (13q14.11) - AKAP11 - XM_015363713.1/TNFSF11 – EPSTI1 - DNAJC15** ●
**Danio:**	**DGKH (13q14.11) - AKAP11 - XM_002663351.6/TNFSF11 – EPSTI1 - DNAJC15** ●
**Takifugu:**	**VWA8 (13q14.11) - AKAP11 - XM_003976029.2/TNFSF11 –** IDH1 (2) - MCM3AP (21) ●
**Callorhinchus:**	**DGKH (13q14.11) - AKAP11 - XM_007907178.1/TNFSF11 – EPSTI1 - DNAJC15** ●
**Rhincodon:**	N. a. - **XM_020536060.1/TNFSF11 – EPSTI1** – N. a. ●

TNFSF11 and TNFSF-Fish4 both have adjacent AKAP proteins

TNFSF-Fish4 (Latimeria: unknown chromosome, region corresponding to human Xp11.22-23)

Homo: gene absent **(Xp11.22-23: SHROOM4 – *DGKK* - CCNB3 – AKAP4 – CLCN5)**

Latimeria: **CCNB3 – AKAP4 – XM_005992884.2/TNFSF-Fish4 – CLCN5 – SHROOM4** ●

Lepisosteus: **CCNB3 – AKAP4 – XM_006632725.2/TNFSF-Fish4 – CLCN5 – SHROOM4** ●

Danio: **CCNB3 – AKAP4 – NM_131843.2/TNFSF-Fish4 – CLCN5 –** PYGM (11) ●
TRP7 (5) - LOC100133315 (11) - **NM_001042713.1/TNFSF-Fish4-2 – CLCN5** – LEAP2 (5) **[WGD]** ●

Takifugu: **CCNB3** – AKAP11 (13) – **XM_003966845.1 – CLCN5 – SHROOM4** ●

Callorhinchus: MARS2 (5) **– CCNB3 – XM_007894375.1/TNFSF-Fish4 – CLCN5 – SHROOM4** ●

Rhincodon: N. a. - AKAP11 (13) **- XM_020514266.1/TNFSF-Fish4 – CLCN5** – N. a. ●

TNFSF11 and TNFSF-Fish4 both have adjacent AKAP proteins

TNFSF10 (Homo: 3q26.31)

**Homo:**	**ECT2 - NCEH1 - TNFSF10 – GHSR - FNDC3B** ●
**Latimeria:**	**MYH1 (17) - NCEH1 – XM_014491441.1 - GHSR - FNDC3B** ●
**Lepisosteus:**	**PPP2R3A (3q22.2-3) - NCEH1 – XM_015361124.1 - GHSR - FNDC3B** ●
**Danio:**	**FNDC3B (3q26.31) - NCEH1 - NM_001002593.2 – MSL2 (3q22.3) – STAG1 (3q22.3)** ●
**Takifugu:**	{ **PPP2R3A (3q22.2-3) - NCEH1 - NM_001246301.1 – MSL2 (3q22.3) – PCCB (3q22.3)** ●
	EMC9 (14) - **NCEH1 - XM_003968157.2 – MSL2 (3q22.3)** – VIPR2 (7) **[WGD]** ●
**Callorhinchus:**	**TCTEX1D2 (3q29) - PCYT1A (3q29) - XM_007911250.1 – GHSR - FNDC3B** ●
**Rhincodon:**	N. a. - **NM_ 020536855.1 – GHSR** – N. a. ●
